# NRBP1 pseudokinase binds to and activates the WNK pathway in response to osmotic stress

**DOI:** 10.1126/sciadv.adv4636

**Published:** 2025-07-16

**Authors:** Ramchandra V. Amnekar, Toby Dite, Pawel Lis, Sebastian Bell, Fiona Brown, Clare Johnson, Stuart Wilkinson, Samantha Raggett, Mark Dorward, Mel Wightman, Thomas Macartney, Renata F. Soares, Frederic Lamoliatte, Dario R. Alessi

**Affiliations:** MRC Protein Phosphorylation and Ubiquitylation Unit, School of Life Sciences, University of Dundee, Dow Street, Dundee DD1 5EH, UK.

## Abstract

WNK family kinases are regulated by osmotic stress and control ion homeostasis by activating SPAK and OXSR1 kinases. Using a proximity labeling approach, we found that osmotic stress promotes the association of WNK1 with the NRBP1 pseudokinase and TSC22D2/4 adaptor proteins, results that are confirmed by immunoprecipitation, mass spectrometry, and immunoblotting studies. NRBP1 pseudokinase is closely related to WNK isoforms and contains a RΦ-motif–binding conserved C-terminal (CCT) domain, like the CCT domains in WNKs, SPAK, and OXSR1. Knockdown or knockout of NRBP1 markedly inhibited basal as well as sorbitol-induced activation of WNK1 and downstream components. We demonstrate that recombinant NRBP1 can directly induce the activation of WNK4 in vitro. AlphaFold-3 modeling predicts that WNK1, SPAK, NRBP1, and TSC22D4 form a complex, in which two TSC22D4 RΦ-motifs interact with the CCTL1 domain of WNK1 and the CCT domain of NRBP1. Our data indicate that NRBP1 and likely its close homolog NRBP2 function as an upstream activator of the WNK pathway.

## Teaser

NRBP1 scaffolds a complex essential for activating WNK kinases in response to osmotic stress.

## INTRODUCTION

The with-no-lysine (WNK) kinases are regulated by osmotic stress and chloride ions and are distinguished by their atypical catalytic lysine residue positioning (*1*). Under hypertonic stress or conditions of low intracellular chloride, WNK kinases undergo autoactivation through T-loop trans-autophosphorylation. This activation, in turn, leads to the phosphorylation of T-loop residues in two well-characterized downstream protein kinase substrates: STE20/SPS1-related proline-alanine-rich kinase (SPAK; also known as STK39) and oxidative stress responsive 1 kinase (OXSR1; previously known as OSR1) (*2*–*5*).

SPAK and OXSR1 contain a conserved C-terminal (CCT) docking domain that specifically interacts with conserved RΦXV-motifs (where Φ denotes a hydrophobic amino acid) on WNK isoforms, a process essential for their efficient activation (*4*, *6*–*8*). Recent studies have also identified two CCT-like domains within WNK isoforms, designated CCTL1 (residues 481 to 571 on WNK1) and CCTL2 (residues 1115 to 1195 on WNK1) (*9*–*12*). While in vitro interactions have been observed between the CCTL1 domain of WNK1 and RΦ-motif peptides (*10*, *11*), the physiological roles of these CCT-like domains in regulating the WNK signaling pathway remain unclear.

Key substrates of SPAK and OXSR1 include a group of electroneutral cation-chloride cotransporters that regulate intracellular chloride influx, cell volume, osmosensing, and blood pressure (*2*, *13*–*17*). These cotransporters include the ubiquitously expressed Na-K-Cl-cotransporter 1 (NKCC1), as well as the kidney-specific Na-Cl cotransporter (NCC) and Na-K-2Cl cotransporter 2 (NKCC2). All of these are phosphorylated and activated by SPAK and OXSR1 (*4*, *17*–*20*). NKCC1, NCC, and NKCC2 each contain conserved RΦ-motifs that bind to the CCT domain of SPAK and OXSR1. This interaction is essential for the efficient phosphorylation and activation of these ion cotransporters (*4*, *6*). Thus, the CCT domain is crucial not only for the activation of SPAK/OXSR1 by WNK isoforms but also for the effective phosphorylation of their substrates.

Additionally, SPAK and OXSR1 also phosphorylate and inhibit the activity of four potassium-driven cation-chloride cotransporters (KCC1, KCC2, KCC3, and KCC4), which mediate chloride efflux from cells (*21*–*24*). The reciprocal regulation of electroneutral cation-chloride cotransporters by SPAK and OXSR1, activating chloride influx (NCC and NKCC1/2) and inhibiting chloride efflux (KCCs), ensures precise control of intracellular chloride levels (*2*, *25*).

At the organismal level, the WNK-SPAK/OXSR1-cation chloride cotransporter pathway is crucial for regulating ion reabsorption in the kidney, which plays a vital role in controlling blood pressure (*13*, *17*, *26*, *27*). Mutations that lead to increased levels of WNK1 or WNK4 in the kidney are associated with rare forms of hypertension (*28*, *29*). Recent research has also shown that WNK kinases contribute to a variety of other cellular processes, including autophagy (*30*), cancer (*31*, *32*), neuronal development (*33*), mitosis (*34*), T cell adhesion and migration (*35*), CD4^+^ T cell survival and activation (*36*), as well as inflammasome activation and pyroptosis (*37*, *38*). Recent studies have also implicated WNK1 as a molecular crowding sensor mediating liquid-liquid phase separation and condensate formation to counteract hypertonic stress induced cell shrinkage (*39*). Given the broad roles of WNK isoforms in regulating a variety of cellular processes, we hypothesized that additional key regulators of the WNK signaling pathway likely exist. Early co-immunoprecipitation studies of WNK1 led to the discovery of its key substrates, SPAK and OXSR1 (*3*), and provided evidence that WNK isoforms can form homo- and heterodimers (*40*). Co-immunoprecipitation experiments also confirmed genetic findings that WNK4 interacts with the KLHL3 and CUL3 E3 ligase components, which regulate intracellular WNK4 levels (*41*). Although several other proteins, including LINGO-1, SGK1, TAK1, and EMC2, have been reported as WNK1 interactors (*42*–*45*), the functional significance of these remains unclear.

In this study, we used a proximity labeling approach to identify regulators of the WNK1 pathway, a method that has proven successful in uncovering arrays of functional regulators in other signaling pathways (*46*, *47*). Using this strategy, we identified a pseudokinase nuclear receptor binding protein 1 (NRBP1), which is closely related to WNK isoforms, as an interactor. Our studies reveal that NRBP1 and likely its close homolog NRBP2 function as a critical regulator of WNK1 signaling pathway.

## RESULTS

### Osmotic stress induces interaction between WNK1 and NRBP1 pseudokinase

**Fig. 1.**
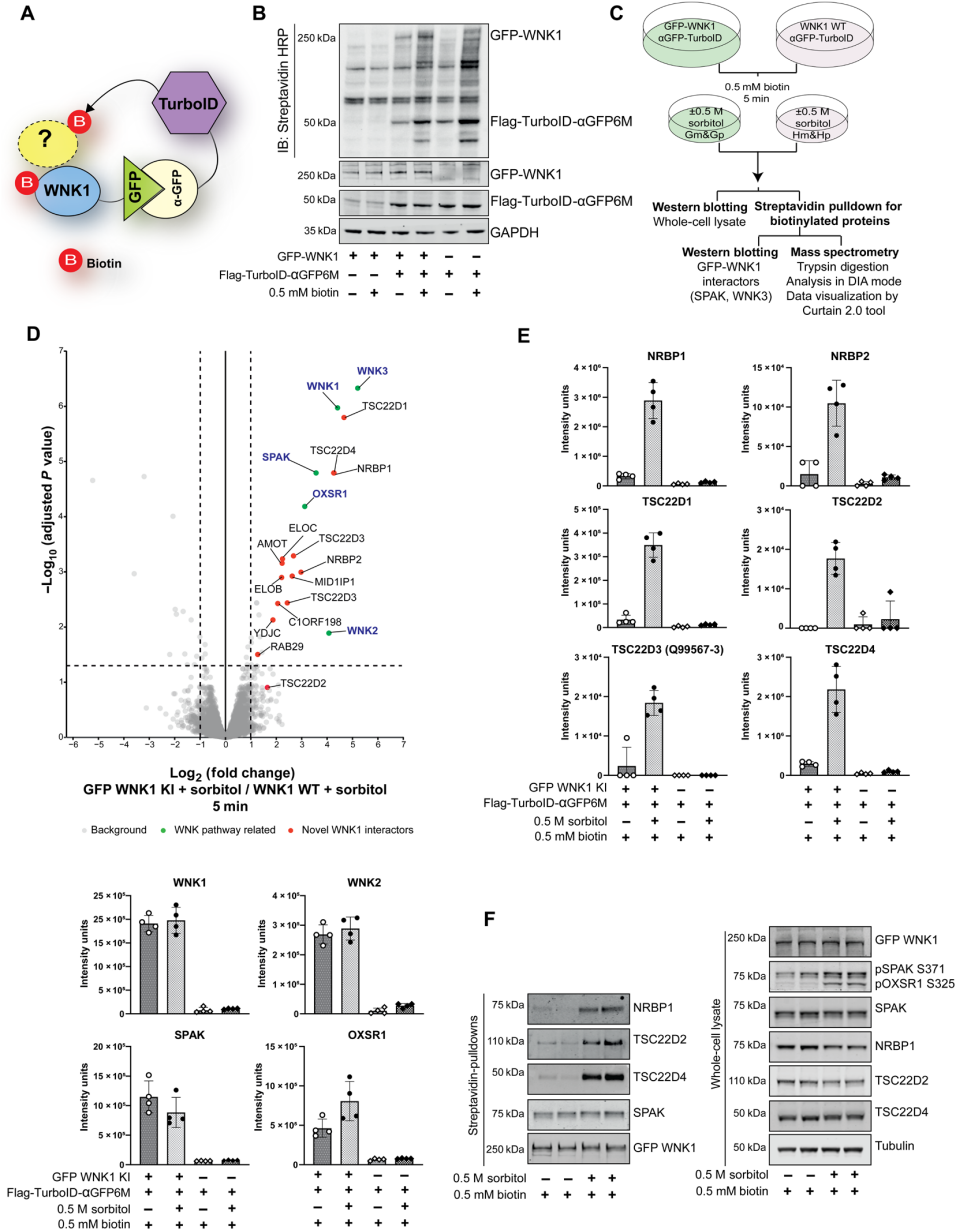
WNK1 is associated with NRBP1, TSC22D2, and TSC22D4 during hypertonic stress. (**A**) Schematic of the GFP-WNK1, FLAG-TurboID-aGFP6M system. (**B**) Immunoblots of the indicated cell extracts with or without FLAG-TurboID-aGFP6M expression and/or exogenous biotin treatment. HRP, horseradish peroxidase; GAPDH, glyceraldehyde-3-phosphate dehydrogenase. (**C**) Schematic of the TurboID-MS experiment under hypertonic stress. GFP-WNK1 knock-in (KI) and wild-type (WT) HEK293 cells expressing FLAG-TurboID-aGFP6M were treated with or without 0.5 M sorbitol for 5 min in the presence of 0.5 mM biotin (*n* = 5 per treatment group). Streptavidin pulldown was followed by washing, S-trap microcolumn peptide preparation, and MS analysis in DIA mode. Data were processed in Python and visualized using the Curtain tool. Note that G denotes GFP WNK1 KI cells and H denotes WT WNK1 cells; m, no sorbitol; and p, with sorbitol. (**D**) Top**:** Volcano plots showing proteins enriched in GFP-WNK1 KI HEK293 cells compared to those in WT HEK293 cells under sorbitol treatment. Proteins highlighted in green include WNK1 and its known interactors (e.g., WNK2, WNK3, SPAK, and OXSR1). Proteins in red represent sorbitol-specific interactors of WNK1. The plots show proteins with ≥2-fold enrichment and statistical significance, normalized to the median intensity of all proteins, with missing values imputed using a Gaussian distribution. *P* values were adjusted using the Benjamini-Hochberg method, with a significance threshold of corrected *P* < 0.05. Data are based on four technical replicates per group. Bottom: Box plots showing the median intensities of notable hits identified in the volcano plot. Curtain link: https://curtain.proteo.info/#/84e40fb8-144e-4859-9b2b-924091194344. (**E**) Box plots of median protein intensities for WNK1 interactors enriched specifically under hypertonic stress conditions. (**F**) Lysates and streptavidin pulldowns from GFP-WNK1 TurboID cells confirm biotinylation of NRBP1, TSC22D2, and TSC22D4, specifically following hypertonic stress.

To identify WNK interactors, we used a proximity labeling approach (*46*, *47*). We generated N-terminal green fluorescent protein (GFP)–tagged WNK1 knock-in (KI) human embryonic kidney (HEK) 293 cell lines stably expressing the Flag-TurboID biotin ligase fused to αGFP6M, an anti-GFP nanobody. This approach is designed to bring the TurboID biotin ligase to GFP-WNK1 and biotinylate proximal proteins (Fig. 1A) and has been used previously to identify potential substrates of the CUL2 E3 ligase diGly receptor KLHDC2 (*48*). We observed that treating GFP-WNK1 KI HEK293 cells expressing Flag-TurboID-αGFP6M with exogenous 0.5 mM biotin for 5 min, before cell lysis, markedly boosted biotinylation of proteins including one corresponding to the molecular weight of GFP-WNK1 (Fig. 1B). These data are consistent with previous work showing that exogenous biotin improves efficiency of TurboID-mediated biotinylation (*47*). We next sought to exploit this system to identify proteins that are in proximity to WNK1 in an osmotic stress–dependent manner. We treated wild-type (WT) and GFP-WNK1 KI cells expressing Flag-TurboID-αGFP6M with 0.5 mM biotin in the presence or absence of 0.5 M sorbitol for 5 min, and biotinylated proteins were enriched by streptavidin affinity purification and analyzed by immunoblot analysis (fig. S1A) and data-independent acquisition (DIA) mass spectrometry (MS) (Fig. 1C and fig. S1B). The phosphorylation of SPAK (pSPAK-S371) and OXSR1 (pOXSR1-S325) confirmed the activation of WNK pathway. We found that WNK pathway components—WNK1, WNK2, WNK3, SPAK, and OXSR1—are biotinylated similarly in the absence or presence of sorbitol (Fig. 1D and fig. S1, C to E). We focused our attention on three interactors that are markedly induced by sorbitol, namely, the pseudokinase NRBP1 (enriched 9.7-fold), and two of its known interactors, namely, transforming growth factor–β (TGFβ)–stimulated clone 22 domain family 2 and 4 (TSC22D2 enriched 3.2-fold and TSC22D4 enriched 6.9-fold) (*49*–*51*). Other TSC22D family members, namely, TSC22D1 (9.8-fold) and TSC22D3 (8-fold) isoforms, were also found to be enriched along with ELOB (6.0-fold) and ELOC (6.7-fold), which are also known NRBP1 interactors (*50*) (Fig. 1, D and E; and fig. S1, C to E). A recent study has also reported co-association of NRBP1, TSC22D family proteins, and WNK1 in biomolecular condensates (*52*). Another recent study that we are coauthors also reports that NRBP1 interacts with the WNK4 isoform of WNK family (*53*). Previous work has shown that NRBP1 comprises a pseudokinase and is devoid of the ability to catalyze phosphorylation as it lacks three critical amino acid motifs in its pseudokinase domain that are essential for kinase activity (DFG, HRD, and VAIK-amino acid sequences) (*54*, *55*). The increased interaction of NRBP1, TSC22D2, and TSC22D4 with GFP1-WNK1 following sorbitol stimulation was also validated by independent immunoprecipitation and immunoblotting studies (Fig. 1F).

### Domain structures of NRBP1, TSC22D2, and TSC22D4

**Fig. 2.**
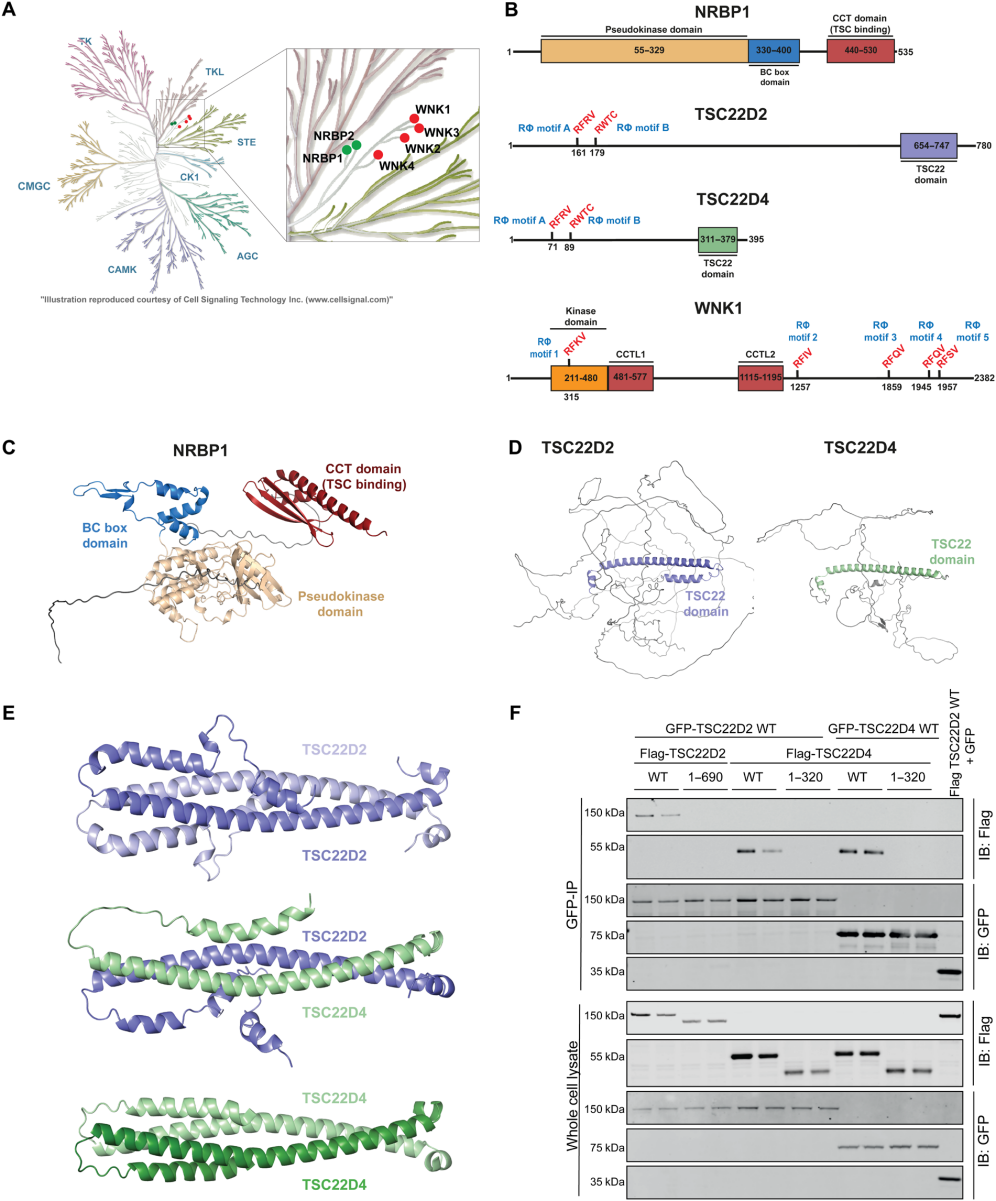
Domain organization of NRBP1, TSC22D2, and TSC22D4. (**A**) Phylogenetic tree displaying evolutionary relationship between WNK kinases (highlighted in red) and the pseudokinases NRBP1 and NRBP2 (highlighted in green). (**B**) Schematic representation of the domain organization of human NRBP1, TSC22D2, TSC22D4, and WNK1 proteins. Key domains include the TSC-TGFβ1–stimulated clone 22 domain (TSC-TGFβ1), BC box (Elongin B and C binding domain), Src homology (SH) domain, and CCT domains. The Rϕ motifs (critical for interaction) are highlighted in red, with two motifs in TSC22D2/4 and five motifs in WNK1. (**C** and **D**) Predicted structural models of full-length NRBP1 and TSC22D2/4 proteins generated using AF3. (**E**) AF3-predicted structures of TSC22D2/4 homo- and heterodimers, focusing on the highly structured C-terminal TSC22 domain. Only the TSC22 domains are depicted, showing multiple contact sites critical for dimerization. (**F**) Co-immunoprecipitation assay in HEK293 cells co-transfected with GFP-TSC22D2/4 full-length (FL) WT and C-terminal deletion mutants of FLAG-TSC22D2. TSC22D2Δ refers to the TSC22D2 mutant lacking residues C-terminal to N690, while TSC22D4Δ lacks residues C-terminal to N320. Immunoprecipitation and immunoblotting confirmed homo- and heterodimerization of the full-length and mutant proteins. The experiment was performed in two biological replicates (*N* = 2), each having two technical replicates (*n* = 2).

The pseudokinase domain of NRBP1 is phylogenetically closely related to WNK1 (*56*, *57*), and potentially NRBP1 could have been termed WNK5 (Fig. 2A). Earlier work has also indicated that TSC22D2 and TSC22D4 play a role in protection from hypertonic stress (*58*). Furthermore, the DepMap database suggests that there is a notable codependence in cell viability between WNK1, NRBP1, and TSC22D2 in certain cancer cell lines, consistent with these components operating within a common pathway (*59*, *60*), and this was also highlighted in another recent study (*52*).

NRBP1 (535 residues) has a pseudokinase domain at its N terminus, followed by a BC box–binding domain known to interact with Elongin B and Elongin C, components of the Cullin 2 and Cullin 4A E3 ligase ubiquitin system, and a C-terminal globular domain (Fig. 2, B and C) (*51*, *61*). AlphaFold-3 (AF3) (*62*) tertiary structure prediction analysis indicates that the C-terminal domain of NRBP1 is similar to the CCT domains found on WNK as well as SPAK and OXSR1 isoforms (Fig. 2C and fig. S2). This observation was also noted in another recent study (*57*). The AF3-predicted tertiary structure of TSC22D2 (780 residues) and TSC22D4 (395 residues) suggests that most of protein is disordered, apart from a long α helix region lying toward the C terminus, encompassing a region of these proteins referred to as the TSC22 domain. The N terminus of TSC22D2/4 proteins harbors two highly conserved potential RΦXX CCT binding motifs (*49*), designated as RΦ-motif in our study (Fig. 2, B and D). We have termed these as RΦ-motif-A (RFRV) and RΦ-motif-B (RWTC). It should be noted that RΦ-motif-B (RWTC) has a Trp residue rather than a Phe residue present in the canonical RFXV motif. Previous work has suggested that the C-terminal domain regions of TSC22D2 and TSC22D4 form a coiled-coil homo and heterodimerization domain (*63*, *64*), which is supported by AF3 modeling (Fig. 2E) (*65*). Consistent with this, we observed that deletion of the TSC22 C-terminal domain of either TSC22D2 or TSC22D4 ablated co-immunoprecipitation with full-length proteins (Fig. 2F).

### Characterizing interaction of NRBP1-CCT domain with TSC22D2/4

**Fig. 3.**
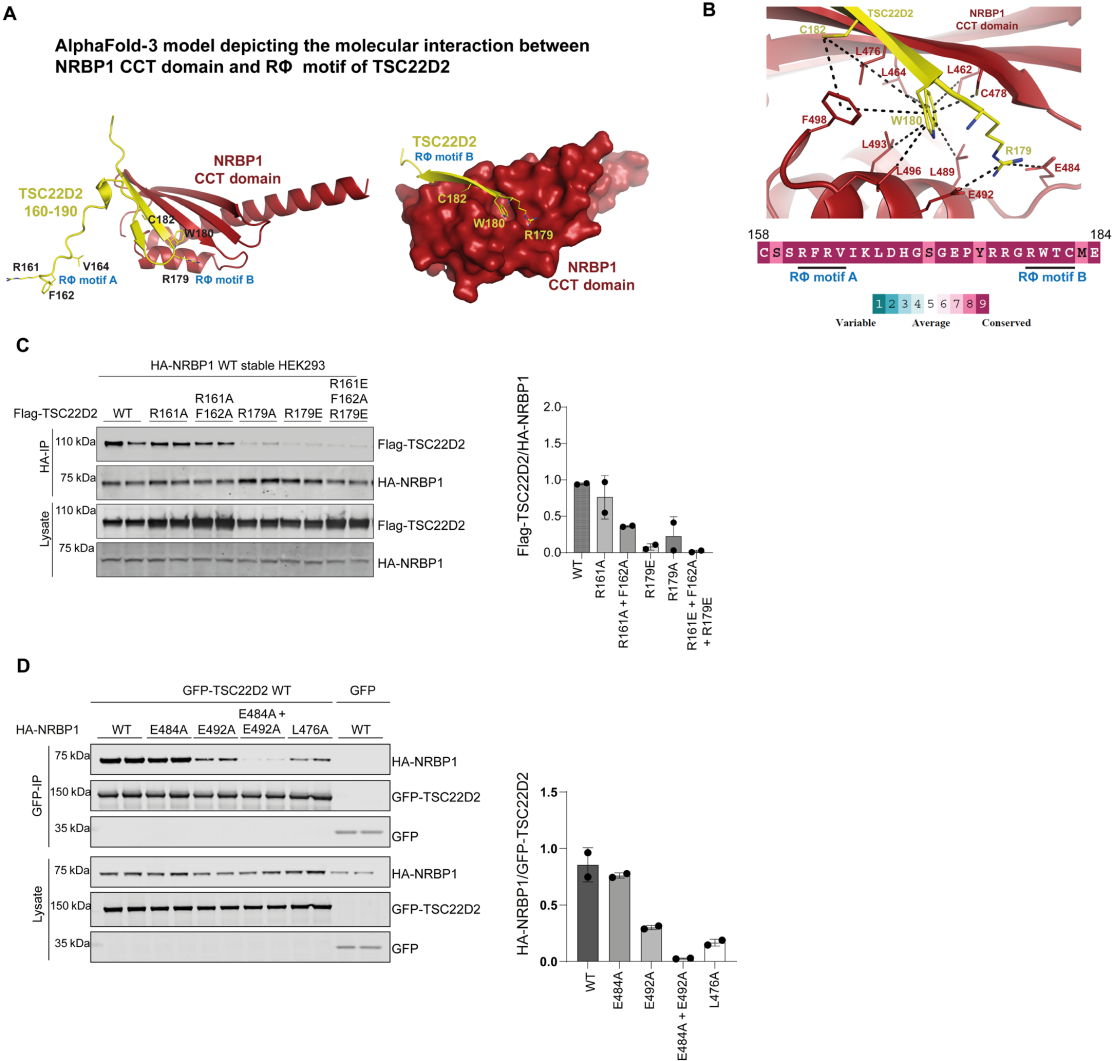
CCT like domain of NRBP1 interacts with the RϕXX motifs of TSC22D2 and TSC22D4. (**A**) Structural modeling using AF3 reveals the molecular interaction between TSC22D2 and NRBP1. Residues 158 to 184 of TSC22D2, containing RΦ motif B, directly interact with NRBP1’s CCT-like domain. (**B**) Two glutamate residues (Glu^484^ and Glu^492^) in the CCT-like domain of NRBP1 form salt bridges with Arg^179^ of TSC22D2’s motif B. Additionally, Trp^180^ of TSC22D2 embeds into a hydrophobic pocket of NRBP1’s CCT domain, involving residues Leu^462^, Leu^464^, Leu^476^, Cys^478^, Leu^489^, Leu^493^, Leu^496^, and Phe^498^. Cys^182^ of TSC22D2 further stabilizes this interaction via hydrophobic interactions with Leu^476^ and Phe^498^ of NRBP1. Conservation analysis using ConSurf (*83*) demonstrates high evolutionary conservation of RΦ motifs A and B, underscoring their functional significance. (**C**) Left: HEK293 cells stably expressing HA-NRBP1 were transfected with FLAG-TSC22D2 mutants harboring alterations in RΦ motifs A and B. After 36 hours, HA immunoprecipitation was performed to assess the interaction between NRBP1 and TSC22D2 mutants. FLAG-TSC22D2 levels in the HA-IP fraction were normalized to those in whole-cell lysates for analysis. Right: Densitometric analysis of the immunoprecipitated samples from two independent experiments (*N* = 2), each performed in duplicate (*n* = 2). (**D**) Left: HEK293 cells were co-transfected with GFP-TSC22D2 WT and HA-NRBP1 WT and the indicated mutants for 36 hours. GFP immunoprecipitation was performed to assess the interaction between TSC22D2 and NRBP1 mutants. HA-NRBP1 levels in the IP fraction were normalized to GFP TSC22D2 levels for analysis. Right: Densitometric analysis of the immunoprecipitated samples from two independent experiments (*N* = 2), each performed in duplicate (*n* = 2).

We next explored how NRBP1 might interact with TSC22D2 and TSC22D4 using AF3 analysis (*62*). The data suggested that highly conserved residues of TSC22D2 (158 to 184) (Fig. 3A) and TSC22D4 (70 to 94) (fig. S3A) encompassing the RΦ-motif-A (RFRV) and RΦ-motif-B (RWTC) discussed above could interact with the NRBP1-CCT domain (Fig. 3B). AF3 modeling indicates that the noncanonical RΦ-motif-B (RWTC) in both TSC22D2 (Fig. 3A) and TSC22D4 (fig. S3A) bind to the CCT domain of NRBP1. To further explore this, we mutated the RΦ-motif-A and RΦ-motif-B motifs of TSC22D2 (Fig. 3C) and tested the impact on NRBP1 binding in a HEK293 cell co-expression system. For TSC22D2, consistent with the AF3 modeling, mutation of the RΦ-motif-B (R179A and R179E mutation) markedly affected binding to NRBP1, whereas, in the case of RΦ-motif-A mutants, the binding was reduced to half with the R161A + F162A mutant (Fig. 3C). Previous work undertaken in *Drosophila* demonstrated that fragments of TSC22D2 and TSC22D4 encompassing both RΦ-motifs co-immunoprecipitated with a C-terminal region of NRBP1 encompassing the CCT domain (*49*). To further validate the mechanism by which NRBP1 binds to TSC22D2 and TSC22D4, we also mutated two NRBP1 CCT domain residues (E484A and E492A) predicted by AF3 to interact with the Arg residue of the RΦ-motif-2 of TSC22D2 and TSC22D4 (Fig. 3B and fig. S3B). The ability of the NRBP1(E484A+E492A) mutant to co-immunoprecipitate TSC22D2 and TSC22D4 was markedly reduced compared to that of WT NRBP1 (Fig. 3D and fig. S3B).

### WNK1 phosphorylates NRBP1 on its T-loop residue

**Fig. 4.**
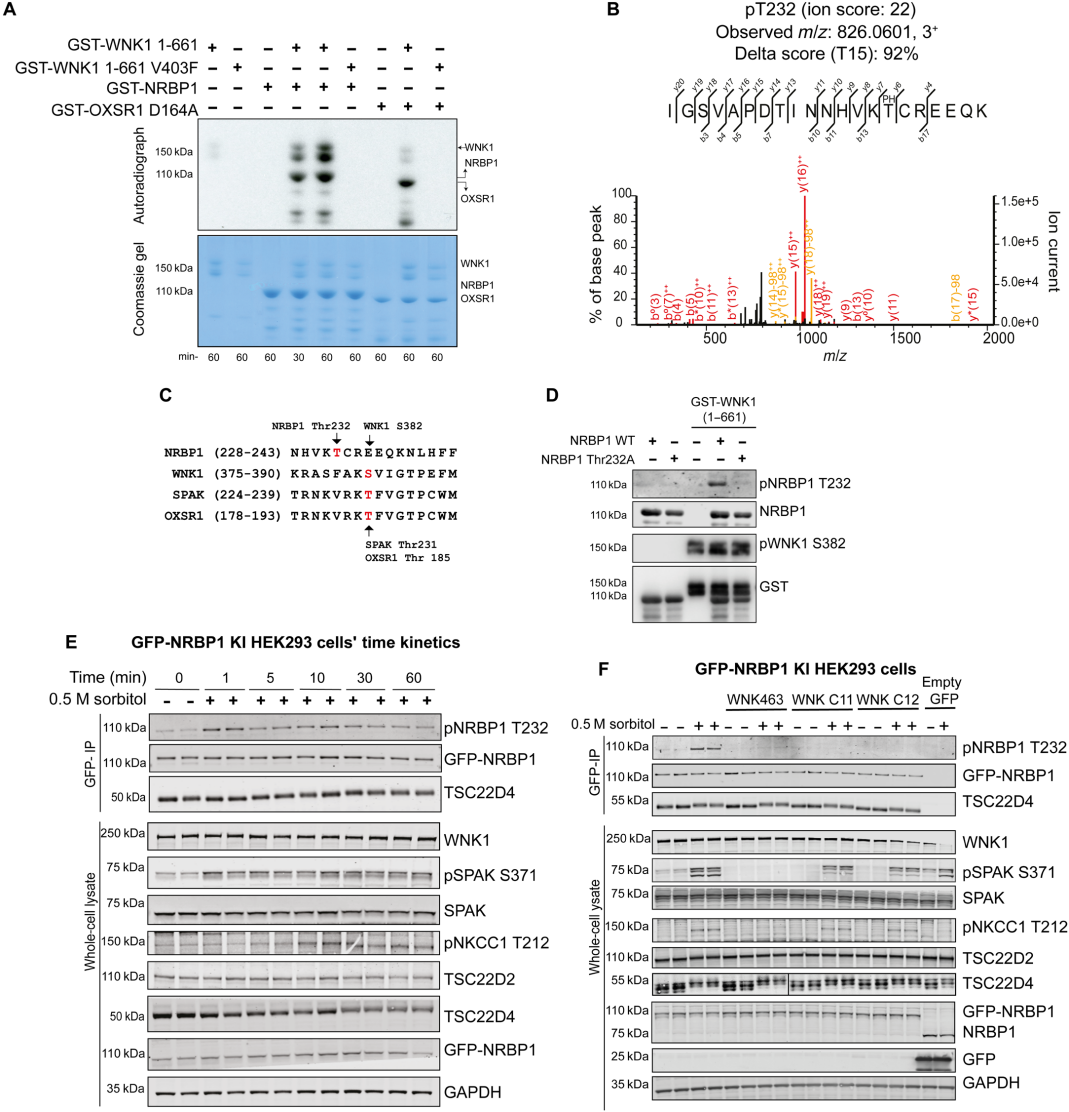
WNK1 directly phosphorylates NRBP1 in vitro and in cells. (**A**) Autoradiograph (top) and Coomassie-stained gel (bottom) showing phosphorylation of NRBP1 by the WNK1 kinase domain (residues 1 to 661) in an in vitro kinase assay. (**B**) MS/MS spectra confirming phosphorylation of the NRBP1 peptide at Thr^232^, the putative T-loop phospho-acceptor residue. Data are from three technical replicates for each condition. (**C**) Protein sequence alignment of T-loops from NRBP1, SPAK (STK39), and OXSR1 (OXR1), highlighting Thr^232^ of NRBP1 as a conserved phosphosite. The alignment was performed using MUSCLE (*84*) and visualized with Jalview (*85*), demonstrating motif similarity to other known T-loop phosphorylation sites. (**D**) Immunoblot analysis using a sheep phospho-specific NRBP1 Thr^232^ antibody confirms phosphorylation in a kinase assay with GST-tagged NRBP1 (WT) and a Thr^232^A mutant by recombinant GST-WNK1 kinase domain (2 to 661). (**E**) Immunoblot analysis of GFP-NRBP1, TSC22D2, and TSC22D4 immunoprecipitated from GFP-NRBP1 KI HEK293 cells treated with 0.5 M sorbitol for various time points. Whole-cell lysates were also analyzed to assess phosphorylation dynamics. Data represent two independent experiments, each with two technical replicates. (**F**) Immunoblot analysis showing the impact of WNK463 (pan-WNK inhibitor) and WNKC11/12 (WNK1/3-specific inhibitors) on NRBP1 phosphorylation and the broader WNK pathway. Both whole-cell lysates and immunoprecipitated NRBP1 complexes were analyzed. For (E) and (F), representative blots from one experiment are shown, with two independent experiments performed, each with two technical replicates.

**Fig. 5.**
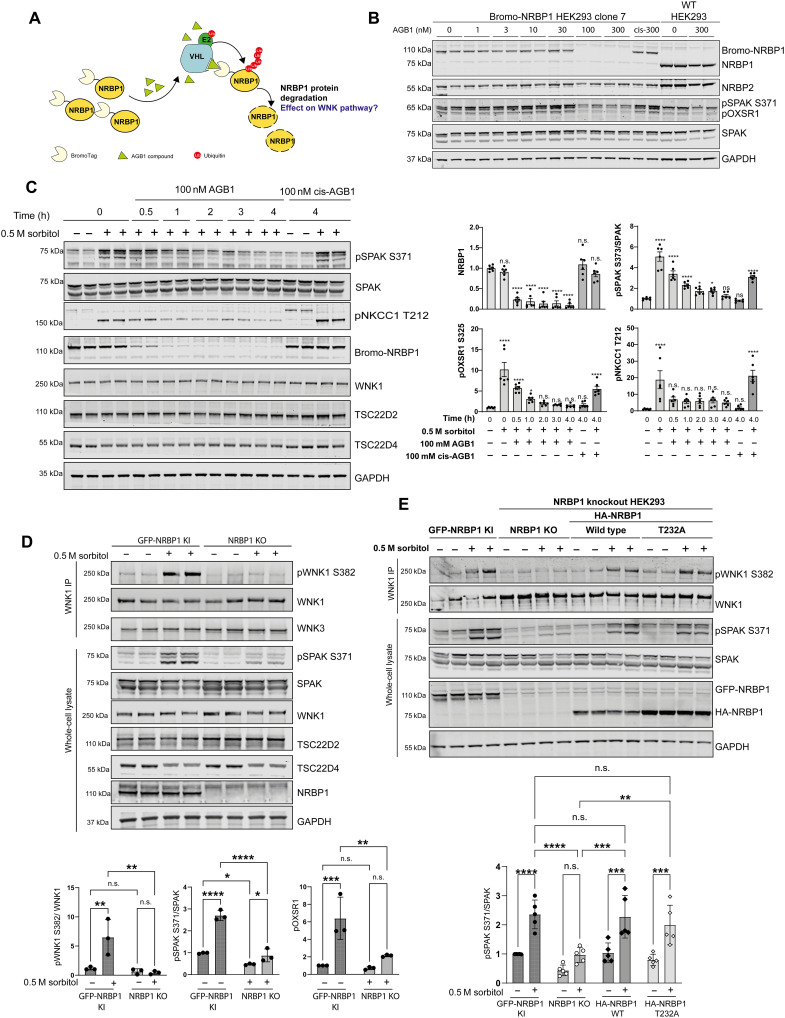
NRBP1 regulates the WNK1 pathway. (**A**) Cartoon illustration depicting the inducible degradation system used for NRBP1. NRBP1 was N-terminally tagged with the bdTag (BromoTag), which, upon binding to the AGB1 compound, is directed to the proteasome for degradation. (**B**) Bromotag-NRBP1 KI HEK293 cells were treated with increasing concentrations of AGB1 (active compound) or cis-AGB1 (negative control inactive compound) for 3 hours. Lysates were subjected to immunoblotting to assess dose-dependent degradation of NRBP1. (**C**) Left: Time-dependent degradation of NRBP1 was analyzed alongside the WNK1 signaling pathway. BromoTag-NRBP1 KI HEK293 cells were treated with 100 μM AGB1, followed by 0.5 M sorbitol for 30 min. The impact on WNK pathway markers was assessed by immunoblotting. Right: Densitometric analysis of immunoblot results showing the changes in WNK pathway activation. Data represent the result of three independent experiments (*N* = 3), each with two technical replicates (*n* = 2). h, hours. (**D**) Top: The impact of NRBP1 knockout on WNK1 activation (pWNK1-S382) was assessed by immunoprecipitating endogenous WNK1 and immunoblotting. Bottom: Densitometric analysis of the western blots were performed. Data represent the result of three independent experiments (*N* = 3), each with two technical replicates (*n* = 2). (**E**) The impact of NRBP1 knockout and rescue with either WT NRBP1 or the Thr^232^A mutant was assessed on the WNK pathway by immunoblot analysis. Bottom: Densitometric analysis of the western blots was performed**.** Data represent the result of three independent experiments (*N* = 3), each with two technical replicates (*n* = 2). For (C) to (E), statistical analysis was performed using two-way analysis of variance (ANOVA) with Šidák’s multiple comparisons test (**P < 0.01; ***P < 0.001; ****P < 0.0001). n.s., not significant.

We next explored whether recombinant WNK1 could phosphorylate NRBP1. We incubated recombinant WT and kinase inactive (WNK1 V403F mutant) WNK1 (residues 1 to 661) with either full-length NRBP1 or kinase inactive OXSR1 (D164A) (all proteins expressed in *Escherichia coli*) in an in vitro Mg(γ-^32^P)ATP (adenosine 5′-triphosphate) phosphorylation reaction for 30 and 60 min and monitored phosphorylation following ^32^P-autoradiography of a Coomassie-stained gel. We observed that WT WNK1 phosphorylated NRBP1 to a similar extent as kinase inactive OXSR1, a physiological substrate of WNK1 (Fig. 4A). No phosphorylation of NRBP1 or OXSR1 was observed with kinase inactive WNK1. Our data also suggested that the autophosphorylation of WNK1 was enhanced by NRBP1 (Fig. 4A), and this is discussed further in Fig. 5. Trypsin digestion and MS analysis of the glutathione *S*-transferase (GST)–NRBP1 revealed a phosphorylated peptide encompassing the T-loop of the pseudokinase domain, phosphorylated at Thr^232^, which was not observed with the kinase dead WNK1 (Fig. 4B). A ClustalW sequence alignment indicate that the T-loop NRBP1-Thr^232^ residue is not conserved in WNK1, SPAK, or OXSR1 and lies three residues before the WNK1 (Ser^382^), SPAK (Thr^231^), or OXSR1 (Thr^185^) T-loop sites (Fig. 4C). The Thr^232^ site in NRBP1 is conserved in evolution from mammals to frog and fish, but not in Fly (fig. S4A). We generated a sheep polyclonal NRBP1 phospho-specific Thr^232^ antibody whose specificity was verified by showing that it detected in immunoblot analysis WT NRBP1, but not NRBP1(Thr^232^A), after in vitro phosphorylation with WNK1 and Mg-ATP (Fig. 4D).

As our NRBP1 phospho-specific pThr^232^ was not sufficiently sensitive to detect phosphorylated NRBP1 in the whole-cell extract, we generated N-terminal–tagged GFP-NRBP1 KI HEK293 cells to permit facile immunoprecipitation of the endogenous NRBP1 complex using high affinity GFP nanobody, before immunoblotting. A time-course analysis revealed that, following treatment of HEK293 cells with 0.5 M sorbitol, a notable increase in phosphorylation of NRBP1 at Thr^232^ was observed within 1 min that was maintained for 30 min (Fig. 4E). We also noted that sorbitol treatment induced a band shift of co-immunoprecipitated TSC22D4 (Fig. 4E). Our data suggest that the band shifts of TSC22D4 is a result of phosphorylation, as incubation of immunoprecipitated GFP-NRBP1 with lambda phosphatase reduced this band shift (fig. S4B).

We next found that the kinase domain of WNK1 and WNK3 phosphorylated NRBP1 at Thr^232^ in vitro (fig. S4C) and that the pan WNK isoform inhibitor, WNK-463 (*66*), as well as two WNK1/WNK3 selective inhibitors (WNK C-11 and WNK C-12) (*51*), blocked sorbitol-induced phosphorylation of NRBP1 at Thr^232^ in HEK293 cells (Fig. 4F). None of the WNK1 inhibitors affected the band shift in TSC22D4 observed with sorbitol, suggesting that this phosphorylation is mediated independently from WNK isoforms (Fig. 4F).

### NRBP1 interactome ± sorbitol treatment

**Fig. 6.**
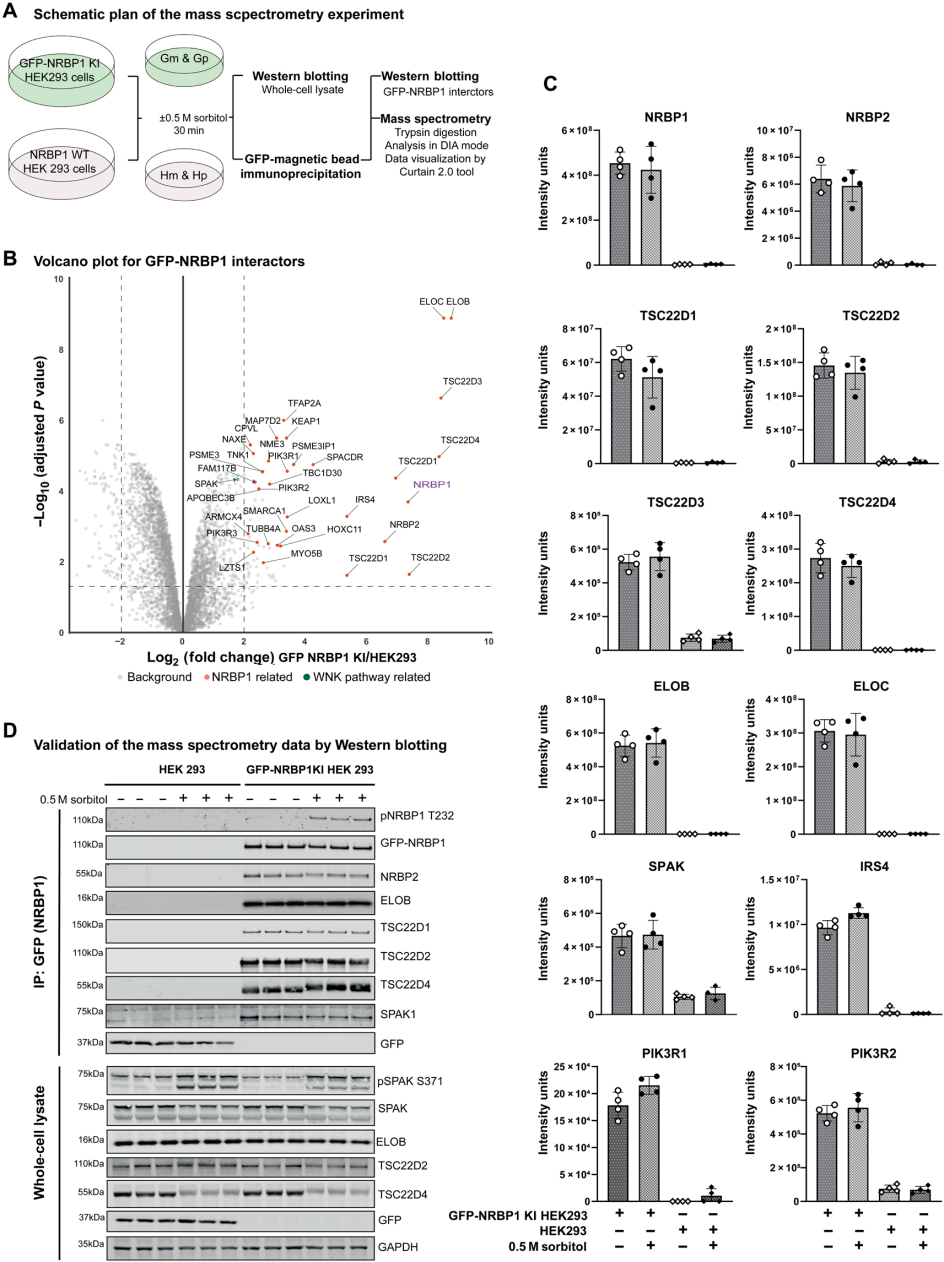
NRBP1 interactors. (**A**) Schematic representation of the MS protocol used to identify NRBP1 interactors under basal and sorbitol stress conditions. GFP-NRBP1 KI HEK293 cells and WT HEK293 cells (negative control) were treated with or without 0.5 M sorbitol for 30 min (*N* = 5 for each group). Following GFP-magnetic bead pulldown and washing, samples were processed for MS using S-trap microcolumns according to the manufacturer’s protocol. Peptides were identified in DIA mode, analyzed in Python, and visualized with the Curtain tool. Note: Gm-GFP NRBP1 cells without sorbitol, Gp-GFP NRBP1 cells with sorbitol, Hm-WT NRBP1 cells without sorbitol, and Hp-WT NRBP1 cells with sorbitol. (**B**) Volcano plot showing proteins significantly enriched (≥4-fold) as NRBP1 interactors under unstimulated conditions. GFP-NRBP1 intensities were normalized to WT-NRBP1, and statistical significance was determined using Benjamini-Hochberg correction. Proteins with corrected *P* values of <0.05 were considered significant. Data from four technical replicates per group were visualized using the Curtain 2.0 tool. Curtain link: https://curtain.proteo.info/#/a38745d4-927f-411e-a89d-b10e6bdd2f9c. (**C**) Box plots showing normalized protein intensities of the interactors identified as significant in the volcano plot (B). (**D**) Selected NRBP1 interactors identified in MS were validated by Western blotting. Experiments were performed twice, with three technical replicates confirming the enrichment of interactors detected in the MS analysis.

To analyze the proteins that associate with endogenous NRBP1, we undertook MS analysis of endogenous GFP-NRBP1 immunoprecipitated from HEK293 cells treated with ±0.5 M sorbitol for 30 min. Cells were lysed in a buffer containing NP-40 (0.5% by volume) and 0.15 M NaCl, and, following immunoprecipitation of GFP-NRBP1, samples were digested with a mixture of trypsin and LysC protease, processed for quantitative data independent acquisition MS and data analyzed through DIA-NN (Fig. 6A) (*67*). Control immunoblotting confirmed successful immunoprecipitation of GFP-NRBP1 along with TSC22D4 and that sorbitol induced phosphorylation of SPAK and upshift of TSC22D4 (fig. S5A). Principal components analysis revealed four distinct groups mapping with the WT control and GFP-NRBP1 cells treated with ±sorbitol (fig. S5B) with notable enrichment of peptides in the GFP-NRBP1 cell lines compared to WT (fig. S5C). We next analyzed proteins that co-immunoprecipitated specifically with GFP-NRBP1 in non–sorbitol-treated cells, with data visualized using the CURTAIN interactive volcano plot software (*68*), in which the data could be viewed and analyzed using the web link provided in the figure legend (Fig. 6B). Proteins enriched >4-fold in the GFP-NRBP1 immunoprecipitates from unstimulated cells were NRBP1, NRBP2, TSC22D1, TSC22D2, TSC22D3, TSC22D4, ELOB, and ELOC and a group of proteins involved in the PI3K signaling system, namely, IRS4, PIK3R1, PIK3R2, and PIK3R3 and not significantly affected by sorbitol stimulation (Fig. 6, C and D). Several other proteins were also enriched to a lower extent in the GFP-NRBP1 immunoprecipitates from unstimulated cells and not affected by sorbitol administration, namely, SPAK, MAP7D2, NME3, SPACDR, TBC1D30, LOXL1, MYO5B, OAS3, TUBB4A, SMARCA1, FAM117B, FAM111B, and HOXC11 (fig. S6).

**Fig. 7.**
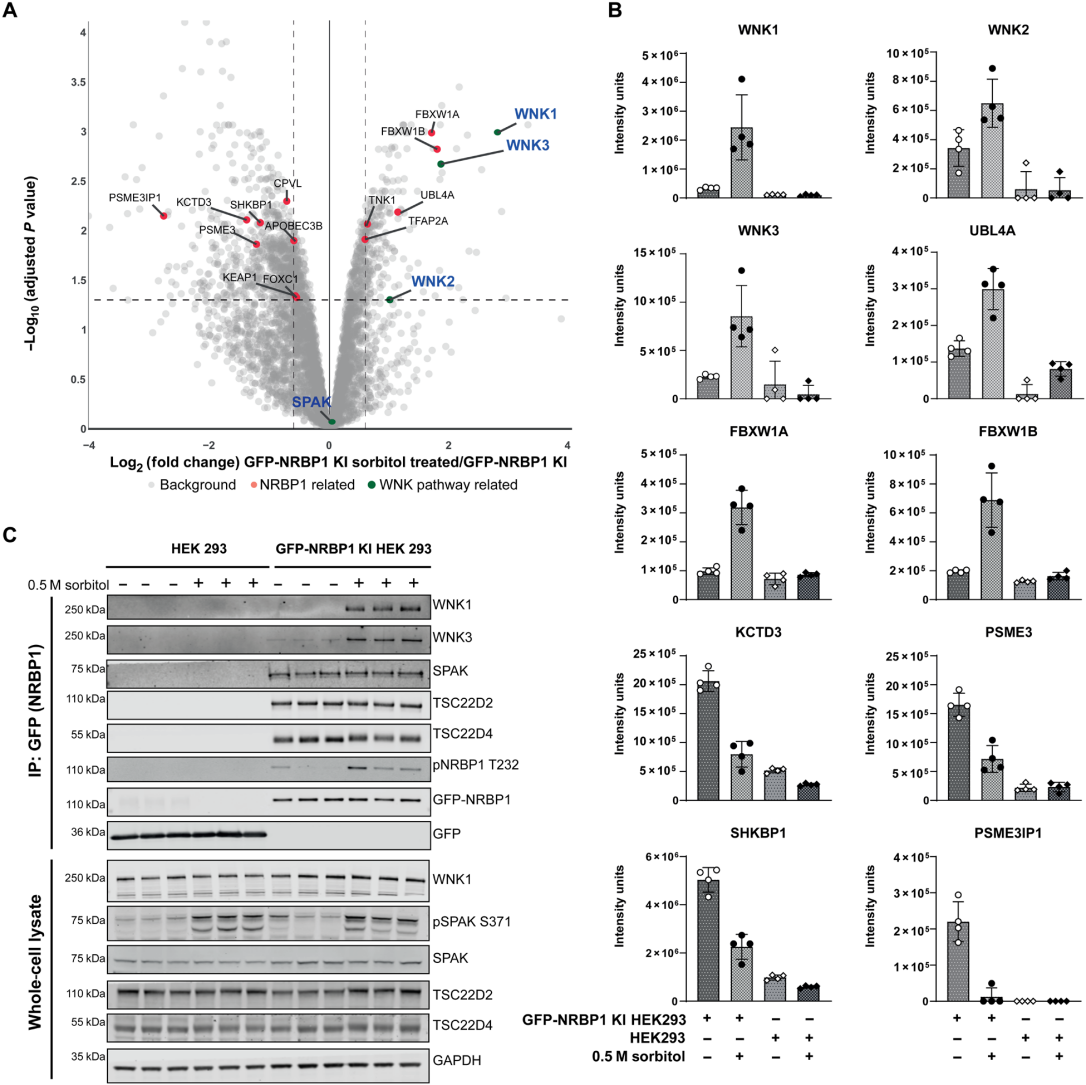
NRBP1 differential interactors. (**A**) Volcano plot displaying NRBP1 interactors significantly enriched (≥2-fold) following treatment with 0.5 M sorbitol. GFP-NRBP1 intensities were normalized to WT-NRBP1 within the sorbitol-treated group. Statistical significance was determined using Benjamini-Hochberg correction, with proteins considered significant at a corrected *P* value of <0.05. Data represent four technical replicates per group, visualized using the Curtain tool. Curtain link: https://curtain.proteo.info/#/6a8a5480-b5db-4c1e-ab94-c7403a37f850. (**B**) Box plots showing normalized protein intensities for the significant hits identified in the volcano plot (A). (**C**) Key NRBP1 differential interactors identified by MS were validated by Western blotting. Experiments were performed twice, with three technical replicates confirming the enrichment of interactors detected in the MS analysis.

Consistent with our proximity labeling data revealing that sorbitol induces the association of WNK1 and NRBP1 (Fig. 1, D and E), we observed that sorbitol stimulation induced significant association of NRBP1 with WNK1 (7-fold) as well as WNK2 (2-fold) and WNK3 (3.5-fold) in addition to three proteins involved in ubiquitin pathways UBL4A (2-fold), βTrCP1/FBXW1A (3.5-fold), and βTrCP2/FBXW1B (3.5-fold) (Fig. 7, A and B). Immunoblotting confirmed that sorbitol induced association of NRBP1 with WNK1 and WNK3 (Fig. 7C). We also observed that several proteins’ (including PSME3, PSME3IP1, SHKBP1, and KCTD3) interaction with GFP-NRBP1 was reduced following sorbitol stimulation (Fig. 7, A and B).

### Rapid degradation or knockout of NRBP1 inhibits WNK activation and phosphorylation of SPAK/OXSR1 and NKCC1

To define the role that NRBP1 plays in regulating the WNK pathway in cells, we first used a targeted protein degradation approach to rapidly degrade NRBP1. We used a CRISPR KI approach to attach a BromoTag, an inducible degron motif (*69*), to the N terminus of NRBP1 in HEK293 cells. Using this approach, the AGB1 heterobifunctional degrader compound hijacks the VHL E3 ligase to induce rapid ubiquitylation and subsequent degradation of the BromoTag-NRBP1 protein (Fig. 5A) (*69*). We observed that doses of 100 or 300 nM AGB1 for 3 hours reduced BromoTag-NRBP1 levels > 95% without affecting NRBP2 levels (Fig. 5B and fig. S7A). Although these cells were not stimulated with sorbitol, knockdown of NRBP1 was accompanied by a marked reduction in levels of basal T-loop phosphorylation of SPAK/OXSR1 (Fig. 5B and fig. S7B). As a control, we used 300 nM of the inactive cis-AGB1 degrader control compound (*69*) that induced no degradation of BromoTag-NRBP1 and also had no effect on the T-loop phosphorylation of SPAK/OXSR1 levels (Fig. 5B). We next treated BromoTag-NRBP1 HEK293 cells with 100 nM AGB1 for between 0.5 and 4 hours followed by 0.5 M sorbitol for 30 min, in the presence of AGB1 (Fig. 5C). Under these conditions, NRBP1 was reduced by ~80% within 30 min and >95% within 1 hour (Fig. 5C). Reduction in BromoTag-NRBP1 levels was also accompanied by a corresponding decrease in the sorbitol-induced T-loop phosphorylation of SPAK/OXSR1, as well as phosphorylation of the SPAK/OXSR1 substrate NKCC1 (Thr^212^/Thr^217^) (Fig. 5C). To confirm these findings, we also generated six independent clones of NRBP1 knockout HEK293 cells and observed that all of these cell lines displayed notably reduced levels of phosphorylation of SPAK/OXSR1 following sorbitol stimulation (fig. S7C).

We also studied the impact of NRBP1 knockout on the sorbitol-induced phosphorylation of the T-loop Ser^382^ site of WNK1. This was achieved by immunoprecipitating WNK1 from WT and NRBP1 knockout cells treated with ±0.5 M sorbitol for 30 min and immunoblotting with a phospho-specific pSer^382^ antibody. The results notably revealed that loss of NRBP1 markedly reduced sorbitol-induced WNK1 Ser^382^ phosphorylation (Fig. 5D).

We next stably reexpressed WT NRBP1 or NRBP1(Thr^232^A) into NRBP1 knockout cells, using a retrovirus system. Expression of WT NRBP1 in the NRBP1 knockout cells restored WNK-mediated T-loop phosphorylation of SPAK/OXSR1 in sorbitol-treated cells (Fig. 5E). Similar rescue of SPAK/OXSR1 phosphorylation was observed following expression of the NRBP1(Thr^232^A), indicating that the impact NRBP1 has on SPAK/OXSR1 T-loop phosphorylation is independent of NRBP1 Thr^232^ phosphorylation (Fig. 5E).

### Recombinant NRBP1 and NRBP2 activates WNK4

**Fig. 8.**
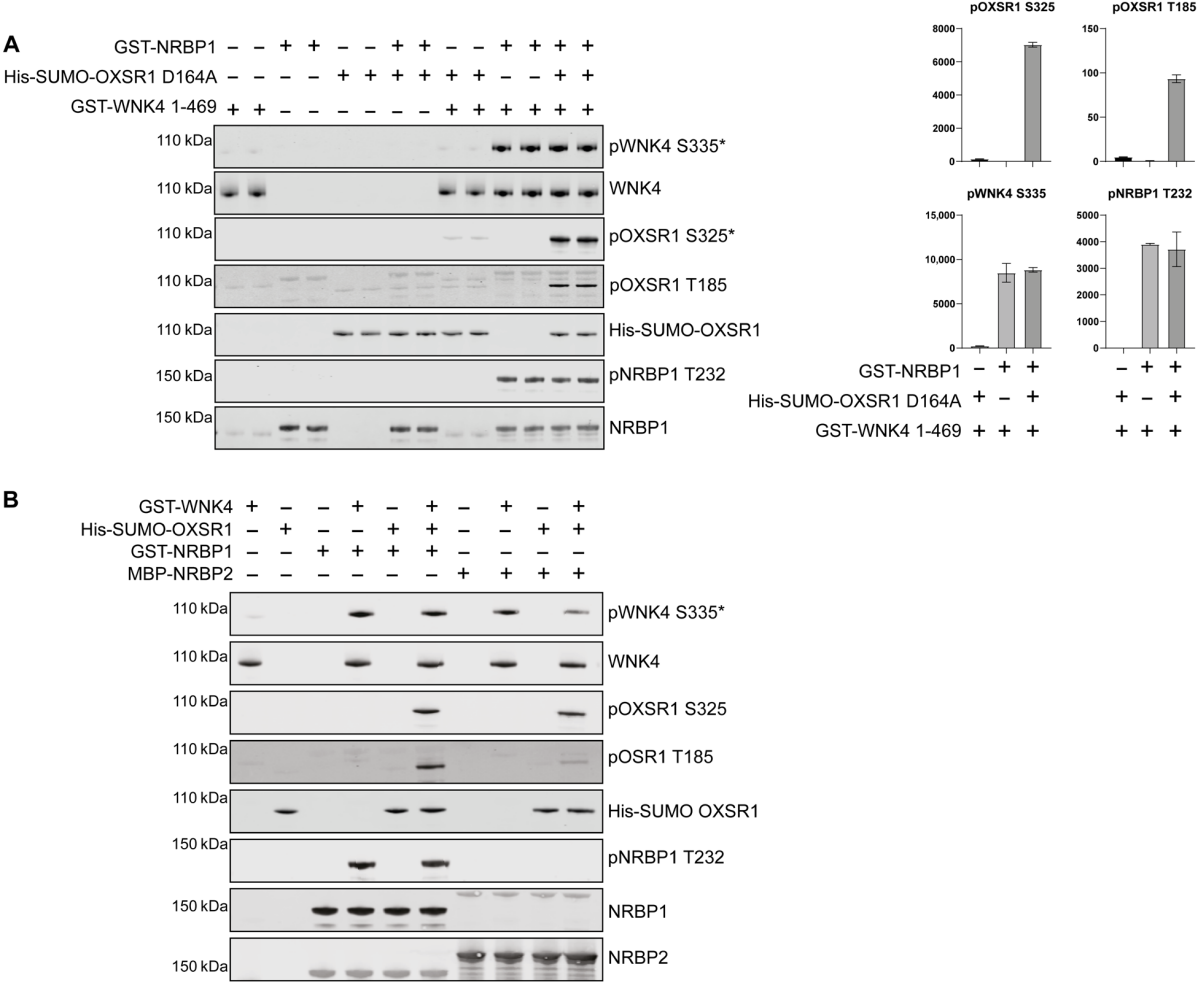
WNK4 is activated in vitro by NRBP1 and NRBP2. (**A**) The activation of WNK4 by NRBP1 was assessed through an in vitro kinase assay. The reaction included GST-WNK4 (residues 1 to 469), GST-NRBP1, and His-SUMO-OXSR1 (D164A, a catalytically inactive mutant). The reaction products were analyzed by immunoblotting to detect WNK4 activation as well as phosphorylation of OXSR1 and NRBP1. Representative results from two independent experiments, each performed with two technical replicates, are shown. Asterisk (*) indicates that the blotting was performed with the pWNK1-S382 and pSPAK-S371 antibody that detects the consensus phosphosite in WNK4 and OXSR1, respectively. (**B**) The activation of WNK4 by NRBP2 was assessed through an in vitro kinase assay using MBP-tagged NRBP2 as mentioned in (A).

We next investigated whether recombinant NRBP1 expressed in *E. coli* could activate WNK isoforms. For these experiments, we used a fragment of WNK4 encompassing the kinase domain (residues 1 to 449) that was expressed in insect cells, as, unlike WNK1, WNK2, and WNK3 isoforms expressed in bacteria, this fragment of WNK4 was not constitutively phosphorylated at its T-loop site (Ser^335^, equivalent to Ser^382^ in WNK1) (*40*). We combined NRBP1 (full length, 2 μM), in the presence or absence of WNK4 (residues 1 to 449, 500 nM), and kinase-inactive OXSR1(D16A) (full length, 2 μM). The mixtures were incubated for 40 min in the presence of Mg-ATP at 30°C. Reactions were stopped with SDS sample buffer and phosphorylation of NRBP1 at Thr^232^, WNK4 at Ser^335^, and OXSR1 at Ser^325^ and Thr^185^ monitored by immunoblotting using phospho-specific antibodies. Consistent with insect cell expressed WNK4 being devoid of activity, in the absence of NRBP1, it only phosphorylated kinase inactive OXSR1 at its T-loop residue to a low level (Fig. 8A). Furthermore, as expected, the NRBP1 pseudokinase did not phosphorylate kinase-inactive OXSR1 in the absence of WNK4 (Fig. 8A). However, by adding NRBP1 to WNK4 and kinase inactive OXSR1, we observed a marked phosphorylation of WNK4, NRBP1, and OXSR1 T-loop residues (Fig. 8A). As NRBP2 interacted with WNK1 in our proximity labeling study and is homologous to NRBP1 and also lacks the canonical residues required for nucleotide binding, we tested its ability to activate WNK4 in vitro. We observed that bacterially produced NRBP2 promoted WNK4 autophosphorylation at Ser^325^ and induced OXSR1 phosphorylation, to a similar extent as NRBP1 (Fig. 8B).

### AF3 analysis of the NRBP1:TSC22D4:WNK1:SPAK complex

AF3 modeling suggests that NRBP1 forms a homodimer via its pseudokinase domain (fig. S8A) and that NRBP1 can also heterodimerize with the kinase domain of WNK1 (fig. S8B). The predicted interaction interfaces in both cases involve extensive surface areas and rely mostly on electrostatics and hydrogen bonds, rather than hydrophobic interactions. Given the size and complexity of these interfaces, it is likely that single-point mutations would be insufficient to disrupt the interaction.

Co-expression and co-immunoprecipitation experiments using differently tagged versions of full-length NRBP1 and NRBP2 provided evidence for the formation of NRBP1:NRBP1 and NRBP2:NRBP2 homodimers, as well as NRBP1:NRBP2 heterodimers (fig. S8C). In addition, a WNK1 fragment comprising the kinase domain (residues 1 to 661) co-immunoprecipitated with NRBP1 in HEK293 cells (fig. S8D), further supporting the modeled interaction.

**Fig. 9.**
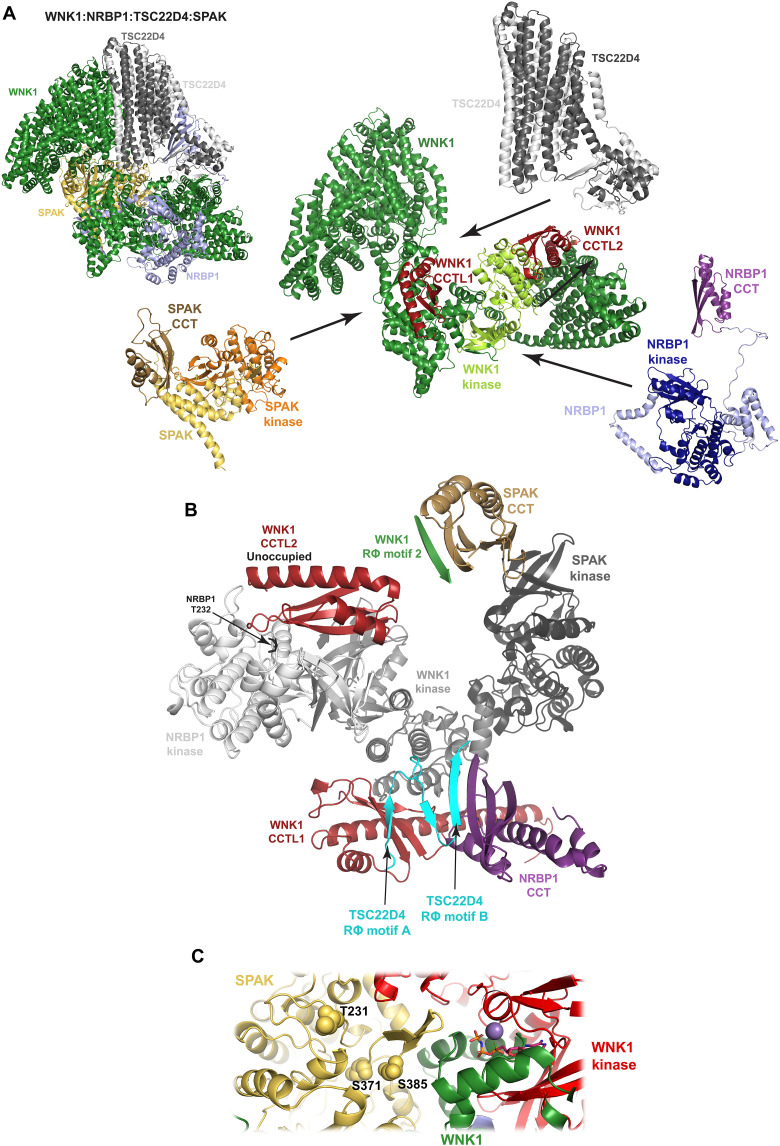
AF3 modeling of the WNK1-SPAK NRBP1 and TSC22D4 complex. (**A**) A structural model of WNK1 in complex with SPAK, NRBP1, and a TSC22D4 homodimer, generated using AF3. The model is shown both as a complete assembly and disassembly into individual components, highlighting key structural domains. The conserved CCT domains in NRBP1 and TSC22D4 are labeled. (**B**) As in (A), but showing only the kinase domains, CCT domains, and RΦ motifs within the WNK1-NRBP1-TSC22D4 complex. The NRBP1 T232 site has been highlighted in black. (**C**) Detailed view of the active site of WNK1 kinase, showing ATP positioned near key phosphorylation sites on SPAK (Thr^231^, Ser^371^, and Ser^385^).

We next modeled a complex consisting of NRBP1, WNK1, SPAK, and homodimeric TSC22D4. The resulting structure featured an ordered TSC22D4 dimer, in which the RΦ-motif-A of TSC22D4 interacts with the WNK1 CCTL1 domain, while the RΦ-motif-B engages the NRBP1 CCT domain (Fig. 9, A and B). In this model, the CCTL2 domain remains unoccupied.

To validate these interactions, we co-expressed isolated CCTL1 and CCTL2 domains with full-length TSC22D2 and TSC22D4. Consistent with the AF3 predictions, both TSC22D2 and TSC22D4 co-immunoprecipitated with CCTL1, and this interaction was abolished by mutating the RΦ-motif-A, but binding was not affected by the RΦ-motif-B mutation (fig. S8, E and F). The isolated CCTL2 domain also co-immunoprecipitated efficiently with full-length TSC22D2 and TSC22D4, but binding was not affected by mutations that ablate either the RΦ-motif-A or RΦ-motif-B motif. These data suggest that both the CCTL1 and CCTL2 domain bind to TSC22D2 and TSC22D4, and further work is required to characterize the CCTL2 binding site and functional role that this plays.

In the AF3 model of full-length WNK1, most non-catalytic regions are predicted to be unstructured, except for the kinase domain and the CCTL1 and CCTL2 domains (fig. S8G). In the context of the model of the full NRBP1:WNK1:SPAK:TSC22D4 complex, these previously disordered regions of WNK1 become ordered (Fig. 9, A and B). Consistent with earlier experimental findings (*4*), the RΦ-motif-2 of WNK1 (residues 1257 to 1260, RFIV) is predicted to interact with the SPAK CCT domain (fig. S8, H and I). The AF3 model of how WNK1 RFXV residue 1257 to 1260 motif binds to the SPAK CCT domain and the structure resembles that of the experimentally determined crystal structure of OXSR1 CCT domain complexed to a WNK4 RFXV motif peptide (Protein Data Bank 2V3S**)** (*7*).

In this model of the NRBP1:WNK1:TSC22D4:SPAK complex, the γ-phosphate of ATP bound to WNK1 is oriented toward three residues in SPAK—Thr^231^, Ser^371^, and Ser^385^—which correspond to the previously reported WNK1 phosphorylation sites (Fig. 9C) (*2*). In the AF3 model of the NRBP1:WNK1:TSC22D4:SPAK complex, Thr^232^ of NRBP1 lies distal to the WNK1 kinase domain, and, therefore, this model does not explain how WNK1 phosphorylates NRBP1 at Thr^232^ (Fig. 4).

## DISCUSSION

NRBP1 was first cloned in 2000 as a protein having a “kinase-like” motif and was predicted to bind to nuclear receptors due to the presence of LXXLL motifs at its C terminus (*61*), which, to our knowledge, has not been demonstrated experimentally. Subsequent work reported various roles for NRBP1 including interacting with Rac3 guanosine triphosphatase (*70*), controlling eye development (*71*), and operating as a negative regulator of gene transcription and tumor progression (*72*). NRBP1 is ubiquitously expressed and knockout of NRBP1 in mice results in early embryonic lethality at day E7.5 (*50*). A genetic analysis in *Caenorhabditis elegans* as well as in mice, suggested that NRBP1 influenced proliferation and homeostasis of intestinal progenitor cells and tumor formation (*50*). This study also reported that NRBP1 co-immunoprecipitated with TSC22D2 and TSC22D4 as well as key components of the ubiquitination machinery including Elongin B and Elongin C (*50*) that form a complex that operates as a substrate adaptor for the Cullin-2 and Cullin-5 E3 ligases (*73*). Our data confirm that Elongin B and Elongin C are constitutively associated with NRBP1 in a manner that is not affected by sorbitol (Fig. 6). We found that sorbitol promotes interaction of NRBP1 with WNK1 as well as other ubiquitin pathway components including βTrCP1/FBXW1A and βTrCP2/FBXW1B that function as members of the F-box substrate adaptors of Cullin-1 E3 ligase complex that operates independent of Elongin B and Elongin C (*73*). Another study has suggested that NRBP1 Cullin containing complexes can regulate the ubiquitylation of two transmembrane proteins termed BRI2 and BRI3 that control the activity of α- and β-secretase (*51*). Given the association of NRBP1 with so many ubiquitin pathway components, in future work, it would be important to explore whether NRBP1 could affect ubiquitylation of WNK pathway components. Our current data indicate that knockout or conditional knockdown of NRBP1 does not affect expression levels of WNK1, SPAK, or OXSR1 proteins, as assessed by immunoblotting.

NRBP1 and its close homolog NRBP2 are classified as class 1 pseudokinases as they lack three key amino acid motifs (DFG, VAIK, and HRD) present on all protein kinases that are required for catalysis of protein phosphorylation. NRBP1 was also shown to lack catalytic activity (*55*) and is incapable of binding ATP or cations (*74*). AF3 modeling (Fig. 9) supported by co-immunoprecipitation studies (fig. S8, C and D) suggests that the pseudokinase domain of NRBP1 can homodimerize, heterodimerize with NRBP2, or heterodimerize with the kinase domain of WNK1. Due to the large interface of these dimer interactions, it would be hard to suppress these interactions by simple mutations. Proximity labeling data and co-immunoprecipitation analysis (Fig. 1 and 4) reveal that osmotic stress induces a rapid and robust interaction of NRBP1 with WNK1 (Figs. 1 and Fig. 7), which has also been reported in a recent study (*52*). Further work is required to understand the mechanism by which sorbitol triggers the association of WNK1 and NRBP1, which has been suggested to be driven by intrinsically disordered regions within WNK1 and the TSC22D proteins (*52*). Our data also indicate that sorbitol induces the phosphorylation of TSC22D4 causing an upward electrophoretic mobility shift, which can be reversed by lambda phosphatase treatment, which appears to be independent of the WNK pathway as this is not affected by WNK inhibitors (Fig. 4). Future work is also required to map these phosphorylation sites and to identify the upstream kinases and whether they play any role in controlling the interaction of NRBP1 with WNK isoforms.

Our results indicate that one of the consequences of NRBP1 association with WNK isoforms is phosphorylation of Thr^232^ residue in the T-loop of NRBP1 that becomes rapidly phosphorylated in a manner that is blocked by a pan WNK kinase inhibitor as well as a WNK1/WNK3 dual inhibitor (Fig. 4). Our data indicate that association of NRBP1 to WNK1 occurs rapidly, within 1 min of osmotic stress, and presumably this binding is required for WNK1 to phosphorylate NRBP1 at Thr^232^. Monitoring Thr^232^ phosphorylation could be used as a biomarker for the association of WNK1 with NRBP1. Our finding that mutation of Thr^232^ residue to Ala does not affect the ability of NRBP1 to rescue SPAK/OXSR1 phosphorylation when reconstituted into NRBP1 knockout cells suggests that Thr^232^ phosphorylation is not required for NRBP1 to activate the WNK1 pathway.

Our data point toward NRBP1 controlling the activation of the basal WNK pathway as well as its sorbitol-stimulated activity as both rapid knockdown or knockout of NRBP1 substantially suppressed basal and sorbitol-induced WNK autophosphorylation and the WNK-mediated phosphorylation and activation of SPAK/OXSR1. The loss of WNK pathway activity can be rescued by expression of NRBP1 in knockout HEK293 cells. Our finding that T-loop phosphorylation and activation of a bacterially expressed inactive fragment of WNK4 can be stimulated by NRBP1 in the presence of OXSR1 (Fig. 8) is consistent with NRBP1 functioning as an upstream activator of the pathway. Our recent studies, in which NRBP1 was knocked out in distal convoluted tubule cells of the kidney, also support that this inhibits the WNK4 signaling pathway’s ability to regulate the level and activity of the thiazide sensitive NCC ion cotransporter (*53*). Our data suggest that NRBP1, WNK1, and SPAK/OXSR1, together with TSC22D2/4, could form a complex, in which the conformation of the WNK1 kinase is stabilized in the active conformation, capable of autophosphorylating Ser^382^ in the T-loop residue, a prerequisite to activate WNK isoforms. These data are also consistent with our proximity labeling (Fig. 1), MS (Figs. 6 and 7) and immunoprecipitation data showing that, in cells treated with sorbitol, TSC22D2/4-NRBP1-WNK1-SPAK/OXSR1 could form a complex.

The TSC22D2/4:NRBP1:WNK1:SPAK complex has four CCT domains (NRBP1-CCT, WNK1-CCTL1, WNK1-CCTL2, and SPAK-CCT). AF3 modeling of the complex indicates that three of the four CCT domains are occupied by binding to specific RΦ-motifs with only the WNK1 CCTL2 domain remaining unoccupied. The SPAK-CCT domain is complexed to the WNK1 RΦ-motif-2 (residues 1257 to 1260, RFIV; Fig. 2B) consistent with previous experimental work (*4*). The NRBP1 CCT domain is complexed to the RΦ-motif-B of TSC22D4, whereas the RΦ-motif-A of TSC22D4 is complexed to the WNK1 CCTL1. These CCT–RΦ-motif interactions likely provide important structural integrity to the TSC22D2/4:NRBP1:WNK1:SPAK complex. The finding that the γ-phosphate moiety of ATP within the WNK1 kinase domain on the TSC22D2/4:NRBP1:WNK1:SPAK complex is oriented toward the three well-characterized SPAK phosphorylation sites (Thr^231^, Ser^371^, and Ser^385^), located on two distinct regions of SPAK, provides further evidence to support the reliability of the model. It would be important in future work to undertake experimental structural analysis to better understand the regulation and function of the TSC22D4:NRBP1:WNK1:SPAK complex. How the NRBP1 pseudokinase controls the conformation and activity of WNK kinases is not defined by the AF3 modeling that we have undertaken.

There are two other TSC22D isoform family members, namely, TSC22D1 and TSC22D3, that were picked up in our WNK1 proximity labeling and NRBP1 MS studies. TSC22D1 has the same conserved RΦ-motif-A (RFRV) and RΦ-motif-B (RWTC) present in TSC22D2 and TSC22D4. However, neither of these motifs is present in TSC22D3, but it could form heterodimers with other TSC22D isoforms. A recent study has suggested that TSC22D1 interacts with the CCT domain of NRBP1 (*57*).

Our data suggest that NRBP1 and NRBP2 have overlapping functions, as they are homologous and were both identified in proximity labeling experiments (Fig. 1). Co-immunoprecipitation results indicate that these proteins can form both homo- and heterodimers (fig. S8C) and similarly activate WNK4 in vitro (Fig. 8). Knocking out NRBP1 reduces but does not eliminate SPAK phosphorylation, suggesting that NRBP2 may contribute to the remaining activation. Future studies should investigate whether NRBP2 compensates for NRBP1 in this process. We were unable to generate NRBP1/NRBP2 double-knockout HEK293 cells, implying that these proteins may play an essential redundant role. Further research is needed to determine whether NRBP2 cooperates with NRBP1 in activating the WNK pathway or whether they have distinct functions.

There are several well-characterized examples of pseudokinases interacting with and activating protein kinases. Notable cases include the LKB1 tumor suppressor kinase, which activates AMPK family members and is regulated by the pseudokinases STRADα and STRADβ (*75*). The ability of STRAD isoforms to activate LKB1 is further enhanced by the adaptor protein MO25 (*76*). Another example is ErbB3, which allosterically activates other epidermal growth factor receptors (*77*). In Janus kinases, the JH2 pseudokinase domain modulates the conformation and activity of the adjacent JH1 protein kinase domain (*78*). Similarly, the KSR1/2 pseudokinases bind and regulate the activity of Raf and mitogen-activated protein kinase kinase isoforms (*79*). In each of these cases, the pseudokinase adopts a conformation that mimics the active state of a catalytically competent kinase in response to specific stimuli. This conformational shift enables the pseudokinase to interact with and allosterically activate its associated kinase. NRBP1 and likely NRBP2, working in conjunction with WNK kinases, represent additional examples of this regulatory pseudokinase-kinase signaling module. In future research, it will be crucial to explore whether osmotic stress, possibly through the formation of biomolecular condensates or another mechanism, could induce the NRBP1 pseudokinase to adopt an active conformation capable of interacting with and activating WNK isoforms. Such insights would advance our understanding of how osmotic stress is sensed and how it triggers the activation of the WNK signaling pathway.

In summary, our data suggest that NRBP1 and TSC22D2/4 form a constitutive complex that interacts with WNK isoforms and SPAK/OXSR1 in response to osmotic stress. This interaction appears to promote WNK activation, leading to the phosphorylation and activation of SPAK/OXSR1, as well as the phosphorylation of NRBP1 at Thr^232^. While the functional significance of NRBP1 phosphorylation at Thr^232^ remains unclear, it serves as a useful marker of NRBP1’s association with WNK isoforms. A recent study found that WNK1-mediated SPAK phosphorylation is essential for CD4^+^ T cell activation, survival, and T cell–dependent antibody responses (*36*). Heterozygous loss of NRBP1 reportedly also results in a loss of CD4^+^ T cells, (www.informatics.jax.org/allele/genoview/MGI:5785083?counter=2#behavior_neurological_id), which we hypothesize may be due to a defect in NRBP1-mediated WNK activation. Together, our findings demonstrate that NRBP1 regulates both basal and sorbitol-stimulated SPAK/OXSR1 activation. Additionally, our parallel work has shown that DCT-specific NRBP1 loss leads to reduced SPAK/OXSR1-mediated NCC phosphorylation in mice on a low-potassium diet (*53*). These findings reinforce the critical role of NRBP1 in regulating WNK pathway activity in animals. Further research is needed to clarify how different activation signals are sensed, their link to biomolecular condensates, and how they regulate NRBP1-WNK interactions. It is also important to explore whether NRBP1’s regulation of WNK isoforms is influenced by chloride ion concentrations, a major regulator of the WNK1 system (*80*).

## MATERIALS AND METHODS

### Plasmids

Standard recombinant DNA techniques were used. Constructs for transient transfections were subcloned into pcDNA5-FRT/TO or pCMV5 vectors. For retroviral transfections, the pBABE puro vector was used. All constructs are available for request from the Medical Research Council (MRC) Phosphorylation and Ubiquitylation Unit (PPU) Reagents webpage at MRC PPU Reagents and Services (www.ppu.mrc.ac.uk/) and are listed in Table 1. The unique identifier (DU) numbers listed below provide direct access to the cloning strategies and sequence information.

**Table 1.** List of constructs used in the study. The constructs are available upon request via the MRC PPU Reagents and Services webpage (www.ppu.mrc.ac.uk/), and the DU numbers indicated below provide direct links to data sheets that contain DNA sequence information.

DU number	Construct name
**For transient transfection studies**	
DU68362	pcDNA5D FRT/TO HA NRBP1
DU72784	pcDNA5D FRT/TO HA NRBP1 L476A
DU72766	pcDNA5D FRT/TO HA NRBP1 E484A
DU72783	pcDNA5D FRT/TO HA NRBP1 E492A
DU72782	pcDNA5D FRT/TO HA NRBP1 E484A + E492A
DU68365	pcDNA5D FRT/TO HA NRBP1 Thr^232^A
DU72496	pcDNA5D FRT/TO GFP TSC22D2
DU72456	pcDNA5D FRT/TO GFP TSC22D4
DU80387	pCMV5D Flag TSC22D2
DU76241	pCMV5D FLAG TSC22D2 R161A
DU80408	pCMV5D FLAG TSC22D2 R161A F162A
DU80409	pCMV5D FLAG TSC22D2 R161E F162A
DU80407	pCMV5D FLAG TSC22D2 R179A
DU80407	pCMV5D FLAG TSC22D2 R179E
DU80449	pCMV5D FLAG TSC22D2 R161E F162AR179E
DU61990	pCMV5D FLAG TSC22D2 M1-I690
DU76249	pCMV5D FLAG TSC22D2 M1-690 R161A F162A
DU68295	pCMV5D HA TSC22D2
DU77694	pCMV5D Flag TSC22D4
DU76243	pCMV5D Flag TSC22D4 R71A F72A
DU80403	pCMV5D Flag TSC22D4 R89A
DU61975	pCMV5D FLAG TSC22D4 M1-N320
DU76244	pCMV5D FLAG TSC22D4 M1-N320 R71A F72A
DU80725	pEGFP-C1 WNK1 450-600
DU10316	pEGFP-C1 WNK1 1056-1217
**For protein expression**	
DU43603	GST-WNK1
DU49486	GST-WNK2
DU4409	GST-WNK3
DU2121	GST-WNK4
DU2961	GST-OXSR1 D164A
DU55144	pet15 6HIS SUMO OXSR1 WT FL 1-527
DU68346	pGEX6P1 NRBP1
DU68840	pGEX6P1 NRBP1 Thr^232^A
DU61825	pMEX3Cb MBP-NRBP2
**For CRISPR-mediated knockout and knock-in studies**	
DU29702	pBABED puro FLAG-TurboID-aGFP_6M_
DU69224	pMK NRBP1 Nter KI donor-donor puro BromoTag
DU64963	pX459 NRBP1 Nter KI sgG1 (guide + Cas9 + puro) (used for both GFP NRBP1 KI and BromoTag NRBP1 KI)
DU69003	pMK-RQ NRBP1 Nter GFP donor
DU29702	pBABED puro FLAG-TurboID-aGFP_6M_
DU57149	pWNK1 Nter GFP IRES2 APEX2 V5 donor
DU52054	pBabeD P U6 WNK1 Nter KI sense A
DU52062	pBabeD P U6 WNK1 Nter KI antisense A
**For retroviral transfections**	
DU77418	pBabeD puro HA NRBP1
DU77419	pBabe Puro HA NRBP1 Thr^232^A

### Antibodies

#### Primary antibodies for Western blotting

Anti-NRBP1: DA204 (MRC PPU Reagents and Services, sheep polyclonal, 1 μg/ml); anti-NRBP2: DA240 (MRC PPU Reagents and Services, sheep polyclonal, 1 μg/ml); anti-TSC22D2: DA056 (MRCPPU Reagents and Services, sheep polyclonal, 1 μg/ml); anti-TSC22D4: DA057 (MRC PPU Reagents and Services, sheep polyclonal, 1 μg/ml); anti-phospho-OXSR1 Thr^185^: PAB29214 (rabbit monoclonal,1:3000); anti-His: ab49936 (mouse polyclonal, 1:5000); anti-phospho-NRBP1 Thr^232^: DA079 (MRC PPU Reagents and Services, sheep polyclonal, 1 μg/ml; with 10 μg/ml non–phospho-peptide); BromoTag antibody: SA599 (MRC PPU Reagents and Services, sheep polyclonal, 1 μg/ml); anti-TSC22D1: 101501-T36 (Sino Biological, rabbit polyclonal, 1:2000); anti-phospho-WNK1 Ser^382^: S099B (MRC PPU Reagents and Services, sheep polyclonal, 1 μg/ml; with non–phospho-peptide at 10 μg/ml); anti-WNK1: Abcam, 37687, 1:2000; anti–phospho-SPAK Ser^371^: S670B (MRC PPU Reagents and Services, sheep polyclonal, 1 μg/ml; with non–phospho-peptide at 10 μg/ml); anti-SPAK: S637B (MRC PPU Reagents and Services, sheep polyclonal, 1 μg/ml); anti-phospho-NCC Thr^53^: PA5-95674 (Thermo Fisher Scientific, rabbit polyclonal, 1:1000; can cross-react with NKCC1 Thr^212^); anti-NRBP1: SAB1408678 (Sigma-Aldrich, rabbit polyclonal, 1:1000); anti-WNK4: NB600-284 (Novus Biologicals, rabbit polyclonal, 1:2000); anti-HA (hemagglutinin): Roche 3F10 (rat monoclonal, 1:3000); anti-FLAG: (mouse monoclonal, 1:3000); anti-GFP: 3H9 (ChromoTek, rat monoclonal, 1:3000); glyceraldehyde-3-phosphate dehydrogenase: Sc-32233 (mouse polyclonal, 1:5000); and α-tubulin: 3873 (Cell Signaling Technology, mouse monoclonal, 1:10,000). All antigen information for both total and phospho-specific antibodies raised in the MRCPPU Reagents and Services is available on its website (www.ppu.mrc.ac.uk/).

The specificity of the NRBP1 (residues 1 to 535, human sequence, DA204), NRBP2 (residues 1 to 509, human sequence, DA240), TSC22D2 (residues 4 to 780, human sequence, DA056), and TSC22D4 (residues 1 to 395, human sequence, DA057) antibodies raised for this study is shown in fig. S9

### Antibody production

Sheep polyclonal antibodies recognizing total proteins are produced by immunizing sheep with recombinant proteins expressed in *E. coli*. For phospho-specific antibodies, the sheep were injected with a peptide antigen encompassing the phosphorylation site (produced by peptides&elephants), and these peptides were conjugated to carrier proteins, keyhole limpet hemocyanin, and bovine serum albumin, through C-terminal linkage with 6-aminohexanoic acid (Ahx), as outlined in *Antibodies: A Laboratory Manual* by Harlow and Lane (1988; Cold Spring Harbor Laboratory Publications). Sheep were immunized with the antigen and subsequently received up to four booster injections, spaced 28 days apart. Blood samples were collected 7 days after each injection. Antibodies were affinity-purified from the sera using the same phospho-peptides used for immunization. Briefly, serum was heat inactivated at 56°C for 20 min and then filtered through a 0.45-μm filter. The inactivated serum was diluted 1:1 with 50 mM tris-HCl (pH 7.5) containing 2% Triton X-100. The diluted serum was passed through a column containing the antigen-conjugated resin, with the flow-through collected twice and stored at −20°C as a backup. The resin was washed with Peptide Wash Buffer [50 mM tris-HCl (pH 7.5), 0.5 M NaCl] until the absorbance at 595 nm [optical density at 595 nm (OD_595_)] was less than 0.003 to eliminate nonspecific proteins and antibodies. Antibodies were eluted in 1-ml fractions using 50 mM glycine (pH 2.5) into 1.5-ml Eppendorf tubes containing 200 μl of 1 M tris-HCl (pH 8.0) to neutralize the elution buffer. The antibody concentration was determined using the Bradford assay and then dialyzed against 1× phosphate-buffered saline (PBS) overnight. The final concentration was adjusted to above 0.1 mg/ml, and aliquots of the purified antibodies were stored at −20°C.

### Cell culture, transient transfection, and cell lysis

HEK293 cells (American Type Culture Collection, CRL-1573) were cultured in Dulbecco’s modified Eagle’s medium (DMEM; Gibco, 11960–085) supplemented with 10% fetal bovine serum (FBS; Sigma-Aldrich, F7524), 2 mM l-glutamine (Gibco, 25030024), and penicillin-streptomycin (100 U/ml; Gibco, 15140122). Cells were maintained at 37°C in a humidified atmosphere with 5% CO_2_ and regularly tested for mycoplasma contamination. For transient transfections, 10 μl of polyethylenimine (PEI; 1 mg/ml; Polysciences Inc., 24765) was mixed with 2 μg of plasmid DNA in 0.5 ml of Opti-MEM (Gibco, 31985–062). The mixture was vortexed gently for 20 s and incubated at room temperature for 30 min. After incubation, the transfection mixture was added dropwise to the cell culture medium in a 100-cm dish. Cells were treated and lysed 24 to 36 hours post-transfection. Following treatment, cells were lysed directly on the plate using ice-cold lysis/immunoprecipitation buffer [50 mM tris-HCl (pH 7.5), 1% (by volume) NP-40, 100 mM NaCl, 1 mM EDTA, 1 mM sodium orthovanadate, 50 mM sodium fluoride, 5 mM sodium pyrophosphate, 10 mM sodium β-glycerophosphate, and cOmplete Mini EDTA-free protease inhibitor (Merck, 11836170001)] without a prior PBS wash. The same lysis buffer was used for immunoprecipitation. Protein lysates were clarified by centrifugation at 17,500 rpm for 15 min at 4°C, and protein concentration was measured using the Bradford assay.

### Quantitative immunoblotting analysis

For immunoblotting, ~20 to 25 μg of protein lysates and 25% of immunoprecipitated fractions were resolved by electrophoresis on precast NuPAGE 4 to 12% Bis-Tris Midi Gels (Thermo Fisher Scientific) at 120 V using NuPAGE Mops-SDS running buffer (Thermo Fisher Scientific). Proteins were transferred to nitrocellulose membranes (GE Healthcare, Amersham Protran Supported, 0.45 μm) for 90 min at 90 V using a transfer buffer [48 mM tris, 39 mM glycine, 20% (by volume) methanol]. The membranes were blocked with 5% nonfat milk in 0.1% TBS-T for 1 hour at room temperature. Primary antibody incubation was performed either overnight at 4°C or for 1 hour at room temperature. Following primary antibody incubation, membranes were washed three times for 10 min each with 0.1% TBS-T. Secondary antibody incubation was performed for 1 hour with anti-sheep, anti-rabbit, or anti-mouse immunoglobulin G secondary antibodies, which were fluorescently labeled with IR680 or IR800. The immunoblots were imaged using the LI-COR Odyssey CLx Western blot imaging system and quantitatively analyzed using Image Studio Lite software (version 5.2.5)

### Immunoprecipitation assay

Immunoprecipitations were conducted as described previously (*17*). Protein lysates (1 to 4 mg, depending on the experiment) were used for immunoprecipitation with NHS-activated GFP and HA-Sepharose nanobeads from MRC PPU Reagents and Services. Approximately 20% of the reaction was reserved as input or whole-cell lysate. For the immunoprecipitation, 20 μl of 1× PBS-washed bead slurry per 2 mg of lysate was used. The incubation was performed at 4°C for 2 hours with gentle agitation at 20 rpm. Beads were then washed sequentially with 1 ml of immunoprecipitation (IP) buffer, followed by two washes with ice-cold 1× PBS containing 0.02% (by volume) NP-40. Each wash was followed by centrifugation at 1*g* for 3 min. Proteins were eluted from the beads by boiling in 50 μl of 2× NuPAGE lithium dodecyl sulfate (LDS) sample buffer at 60°C for 5 min. The eluate was then filtered using a Spin-X Centrifuge Tube Filter (Costar 8161) and denatured with 1% (by volume) β-mercaptoethanol at 95°C for 5 min.

### Statistical analysis

Statistical analyses were conducted using GraphPad Prism (RRID:SCR_002798, version 9.3.1; http://graphpad.com/). The following tests were used: two-tailed unpaired *t* test, applied for comparisons between two independent groups; and one-way analysis of variance (ANOVA), used for comparisons among three or more groups. Post hoc tests were used where appropriate to determine specific group differences following ANOVA.

### Protein expression

#### Vectors and cells

Transform pGEX6P-1 vectors expressing N-terminal GST-tagged proteins (WNK1 (1 to 661), DU43603; WNK2 (1 to 627), DU49486; OXSR1 (1 to 527); NRBP1 (1 to 535), DU68346; and NRBP1 (1 to 535) T232A, DU68840) into BL21 Codon Plus cells. For GST-WNK3 (1 to 579), DU4631, use autoinduction medium. Culture and induction: Inoculate a 16-hour starter culture into 1 liter of Luria-Bertani (LB) broth with carbenicillin (100 μg/ml). Grow at 37°C with shaking at 200 rpm until OD_600_ reaches 0.8. For GST-WNK3, grow until OD_600_ reaches 2. Induce protein expression by adding 0.05 mM isopropyl-β-d-thiogalactopyranoside (IPTG) and incubate at 18°C with shaking at 200 rpm for 18 hours (24 hours for GST-WNK3).

#### Cell lysis and protein purification

Pellet cells at 4200*g* for 30 min. Resuspend the pellet in 20 ml of ice-cold lysis buffer [50 mM tris-HCl (pH 7.5), 250 mM NaCl, 1% Triton X-100, 1 mM EDTA, 1 mM EGTA, 0.1% β-mercaptoethanol, 0.2 mM phenylmethylsulfonyl fluoride (PMSF), and 1 mM benzamidine]. Lyse cells by brief freeze-thaw cycles at −80°C and sonicate (Branson Digital Sonifier) with 10 15-s pulses at 45% amplitude.

#### Clarification and affinity purification

Centrifuge the lysate at 35,000*g* for 30 min. Incubate the supernatant with 2 ml of pre-equilibrated glutathione (reduced form)–agarose (Abcam) beads at 4°C for 1 hour. Wash the beads thrice with a wash buffer [50 mM tris-HCl (pH 7.5), 250 mM NaCl, 0.1 mM EGTA, and 0.1% β-mercaptoethanol]. Elute the proteins using a wash buffer containing 20 mM glutathione (pH 7.5). Dialyze into 50 mM tris-HCl (pH 7.5), 0.1 mM EGTA, 150 mM NaCl, 0.5 mM Tris(2-carboxyethyl)phosphine (TCEP), and 270 mM sucrose, and store at −70°C. Note: GST-OXSR1 D164A should be stored in 50% glycerol instead of sucrose and kept at −20°C.

#### Baculovirus expression system (WNK4)

Generation and infection: Use pFastBac GST-WNK4 (1 to 469), DU30159, to generate recombinant baculovirus with the Bac-to-Bac system (Invitrogen) as per the manufacturer’s instructions. Infect *Spodoptera frugiperda* 21 (Sf21) cells grown at 27°C. Harvest the cells 48 hours postinfection.

#### Harvesting and purification

Pellet the cells at 500*g* for 5 min (10 min deceleration). Resuspend in ^1^/_10_ of the original culture volume with ice-cold lysis buffer [20 mM Hepes-NaOH (pH 7.5), 0.02 mM EGTA, 0.02 mM EDTA, 270 mM sucrose, 0.1% 2-mercaptoethanol, 0.2 mM PMSF, and 1 mM benzamidine]. Incubate at 4°C on a roller for 20 min. Purify GST-tagged WNK4 (1 to 469) as described above and store at −70°C.

### His-SUMO-OXSR1 expression

#### Expression and induction

Transform pET His-SUMO-OXSR1 (1 to 529) D164A, DU55130, into BL21 Codon Plus cells. Induce expression with 150 μM IPTG.

#### Harvesting and lysis

Resuspend the cell pellet in 20 ml of resuspension buffer [50 mM tris-HCl (pH 7.5), 150 mM NaCl, 2 mM MgCl_2_, 20 mM imidazole (pH 7.5), 1 mM dithiothreitol (DTT), 1 mM Pefabloc, and leupeptin (20 μg/ml)]. Lyse cells by adding NaCl (final concentration, 300 mM), Triton X-100 (0.2%), and glycerol (5%).

#### Purification

Add 2 ml of equilibrated Nickel agarose (Abcam) resin to the clarified lysate. Incubate at 4°C on a roller for 1 hour. Wash with a resuspension buffer containing 5% glycerol and 400 mM NaCl, followed by two washes without imidazole. Elute the His-SUMO–tagged proteins in a wash buffer containing 400 mM imidazole. Dialyze into 50 mM tris-HCl (pH 7.5), 0.1 mM EGTA, 150 mM NaCl, 0.5 mM TCEP, and 270 mM sucrose, and store at −70°C.

### Kinase activity assays

#### Reaction setup and reaction mixture

Prepare a 50-μl reaction mixture in tris-based buffer (pH 7.6) containing 50 mM tris-HCl, 2 mM MgCl_2_, and 200 μM ATP. Incubate at 31°C for 20 min to 1 hour with agitation at 1000 rpm using a ThermoMixer (Thermo Fisher Scientific).

#### Enzymes and substrates

Use GST-tagged kinase domains of WNK1, WNK2, WNK3, and WNK4, as well as GST or His-SUMO–tagged OXSR1, NRBP1 WT, and NRBP1 Thr^232^A. The kinase-to-substrate ratio should be 1:10.

#### Stopping the reaction

Terminate the reaction by adding 12.5 μl of 4× LDS loading buffer with 4 mM β-mercaptoethanol. Heat the samples at 70°C for 5 min to denature the proteins.

#### Phosphorylation detection

For kinase assays to identify NRBP1 as a substrate of WNK1, perform the assay in the presence of 0.1 mM (γ-^32^P)ATP (~500 cpm pmol^−1^) for 2 hours.

### Gel electrophoresis and detection

#### SDS–polyacrylamide gel electrophoresis

Resolve the samples on a 10% bis-tris gel. Run the gel for 2 hours.

#### Gel staining

Stain the gel using Coomassie InstantBlue for 1 hour. Destain with water and scan using an Epson scanner.

#### Gel drying and autoradiography

Dry the gel completely using a gel dryer (Bio-Rad). Expose the dried gel to Amersham Hyperfilm for 16 hours at −80°C for autoradiography to visualize radiolabeled proteins.

### Identification of NRBP1 Thr^232^ phosphosite by LC-MS/MS

#### Kinase assay, sample preparation, and kinase reaction

Perform kinase assays using kinase-active GST-WNK1 (1 to 661) and kinase-inactive GST-WNK1 (1 to 661) V403F. Incubate the reaction for 1 hour. Process 20% of the reaction mixture for MS analysis.

#### Reduction and alkylation

In a low-binding tube, reduce the proteins by adding 100 mM TCEP (final concentration, 5 mM) and incubate at 56°C for 30 min on a ThermoMixer. Cool the tubes to room temperature. Alkylate nonoxidized sulfhydryl groups with 100 mM indole-3-acetic acid [IAA; final concentration, 20 mM; prepared in 40 mM triethylammonium bicarbonate (TEABC) (pH 8.5)] in the dark at room temperature with shaking at 1000 rpm.

#### Denaturation and digestion

Denature the proteins with 10 M urea [final concentration, 1.5 M; prepared in 40 mM TEABC (pH 8.5)]. Digest with 200 ng of trypsin Lys-C and incubate at 30°C for 16 hours without shaking. Acidify the digested peptides by adding 20% trifluoroacetic acid (TFA) to a final concentration of 1%. Store the acidified peptides at −20°C until further analysis.

#### LC separation

Perform peptide separations using a Thermo Dionex Ultimate 3000 RSLC Nano liquid chromatography (LC) system. Use 0.1% formic acid as buffer A and 80% acetonitrile with 0.08% formic acid as buffer B. Load peptides onto a C18 trap column with 3% acetonitrile/0.1% TFA at a flow rate of 5 μl/min. Separate peptides on an EASY-Spray column (C18, 2 μM, 75 μm by 50 cm) with an integrated nano-electrospray emitter at a flow rate of 300 nl/min. Apply a segmented gradient: start at 3% buffer B, increase to 35% over 40 min, then to 95% over 2 min, and hold at 95% for 5 min.

#### MS analysis

Analyze the eluted peptides using an Orbitrap Lumos (Thermo Fisher Scientific) mass spectrometer. Set the spray voltage to 2 kV, RF lens level to 40%, and ion transfer tube temperature to 275°C. Operate in data-dependent mode with 3-s cycles. Perform full scans in the range of 375 to 1500 mass/charge ratio (*m*/*z*) with a nominal resolution of 120,000 at 200 *m*/*z*, AGC (automatic gain control) target set to standard, and a maximum injection time of 50 ms. Select the most intense ions above an intensity threshold of 5000 for higher-energy collision dissociation (HCD) fragmentation. Set HCD normalized collision energy to 30%. Acquire data-dependent MS2 scans for charge states 2 to 7 with an isolation width of 1.6 *m*/*z* and a 30-s dynamic exclusion duration. Record all MS2 scans in centroid mode with an AGC target set to standard and a maximal fill time of 100 ms.

#### Data processing and data analysis

Process the .RAW files using Proteome Discoverer v2.4 (Thermo Fisher Scientific) with Mascot v2.6.2 (Matrix Science) as the search engine. Set precursor mass tolerance to 10 parts per million and fragment mass tolerance to 0.06 Da. Use an in-house database (MRC_Database_1) with trypsin/P as the protease, allowing a maximum of two missed cleavages. Configure variable modifications to include oxidation and dioxidation of methionine and phosphorylation of serine, threonine, and tyrosine. Set carbamidomethylation of cysteine as a fixed modification. Use ptmRS for scoring phosphosite identification, with a mass tolerance of 0.5 Da and consideration of neutral loss peaks. Confirm phosphorylation site localization only if peptides have a Mascot delta score and ptmRS probability score above 80%.

### Lambda phosphatase assay

GFP-NRBP1 was immunoprecipitated from 4 mg of lysate following 30 min of sorbitol treatment. The immunoprecipitated protein was resuspended in 100 μl of assay buffer containing 50 mM Hepes (pH 7.5), 100 mM NaCl, 2 mM DTT, 0.01% Brij, and 2 mM MnCl_2_ and then divided equally into two tubes. To one tube, ~2 μg of Lambda phosphatase (MRC PPU Reagents and Services website) was added. Both tubes were incubated at 30°C for 1 hour with gentle agitation at 1200 rpm. The reaction was stopped by adding 10 μl of 4× LDS loading dye containing 4% β-mercaptoethanol and heating at 90°C for 5 min.

### Generation of stable cell lines using CRISPR-Cas9

For GFP KIs, HEK293 cells were transfected with 1 μg of each plasmid vector encoding antisense and sense guide RNAs targeting NRBP1 or WNK1, along with 3 μg of a GFP donor plasmid. For the generation of the BromoTag NRBP1 KI cell line, 2 μg of the donor plasmid and 1 μg of the antisense guide RNA were used. Transfection was carried out for 24 hours, after which cells were selected with puromycin (2 μg/ml; Sigma-Aldrich, P9620) for 48 hours. The cells were then allowed to recover in fresh medium until they reached confluency. Fluorescence-activated cell sorting was used to isolate GFP-positive cells into single wells of two 96-well plates containing conditioned medium. Surviving cell clones were expanded, and the successful KI of GFP or BromoTag to the N terminus was verified using Western blotting, immunoprecipitation, and DNA sequencing. To knock out NRBP1 from the GFP-NRBP1 KI HEK293 cell line, a CRISPR construct targeting GFP was used. GFP-negative cells were then selected for further analysis.

### Generation of stable cell lines by retroviral transduction

To generate stable cell lines, the following retroviral components were prepared: 6 μg of pBABE vectors [pBABE puro HA NRBP1 (DU77418) or pBABE puro HA NRBP1 Thr^232^A (DU77419)], 3.2 μg of pCMV5-GAG/POL, 2.8 μg of pCMV5-VSV-G, and 20 μl of PEI (1 mg/ml). These components were added to 1 ml of Opti-MEM (Gibco, 31985–062). The mixture was vortexed gently and incubated at room temperature for 20 min. The transfection mixture was then added dropwise to HEK293-FT cells (at ~70% confluency) growing in a 10-cm-diameter cell culture dish. After 24 hours, the medium was changed to a fresh medium. Following an additional 24 hours, the medium containing retroviruses was collected and filtered through a 0.22-μm sterile filter. Target cells (at 60% confluency) were then transduced with the viral supernatant in the presence of hexadimethrine bromide (8 μg/ml; also known as polybrene; Sigma-Aldrich, H9268) for 24 hours. After transduction, the medium was replaced with fresh media containing puromycin (2.5 μg/ml; Sigma-Aldrich, P9620) to select for successfully transduced cells. Selection with puromycin was carried out for 48 hours, after which the cells were maintained in fresh culture medium.

### Activation of the WNK1 signaling pathway by hypertonic stress

To activate the WNK1 signaling pathway, cells were exposed to complete DMEM containing 0.5 M sorbitol (diluted in 1× PBS) for 5 to 30 min, as previously described (*81*). Before this, cells were pretreated for 20 min with either the pan-WNK inhibitor WNK463 (MRC PPU Reagents and Services) or the WNK1/3-specific inhibitor WNK C11/12 (MRC PPU Reagents and Services) at a concentration of 10 μM. Following pretreatment, cells were incubated in the respective treatment medium containing the inhibitors.

### Biotin proximity labeling with TurboID

To activate the biotin-ligase activity of TurboID, exogenous biotin was added to the culture medium as previously described (*47*, *82*). Briefly, the growth medium was aspirated from cell culture dishes and replaced with fresh DMEM supplemented with 10% (by volume) FBS, 2 mM l-glutamine, 500 μM biotin (Sigma-Aldrich, B4501), and, optionally, 0.5 M sorbitol (Sigma-Aldrich, S1876). After 5 min, the medium was aspirated, and cells were washed rapidly with ice-cold 1× PBS three times. Cells were then lysed in buffer containing 50 mM tris (pH 7.5), 0.27 M sucrose,150 mM NaCl, 1 mM EDTA, 1 mM EGTA, 1% (by volume) NP-40, 1 mM sodium vanadate (NaVO_4_), 5 mM sodium pyrophosphate (Na_4_P_2_O_7_), 50 mM sodium fluoride (NaF), 10 mM β-glycerophosphate, and a 1× EDTA-free protease inhibitor cocktail (Merck, 11873580001). Lysates were incubated on ice for 10 min and then clarified by centrifugation at 30,000*g* for 10 min at 4°C. The supernatant (soluble cell extract) was collected in a fresh Eppendorf tube and snap frozen in liquid nitrogen and stored at −80°C. The protein concentration was estimated by the Bradford protein assay (Pierce, 23236). We used a TurboID variant known for its substantially improved biotinylation kinetics, reducing labeling times compared to BioID and eliminating the need for hydrogen peroxide treatment to induce activation (*47*, *82*).

### Streptavidin enrichment of biotinylated proteins from cell lysate for LC-MS analysis

For LC-MS analysis, 3.5 mg of cell lysate was used for immunoprecipitation with 25 μl of streptavidin magnetic beads (Pierce, 88817) for 2 hours at 4°C with gentle rotation. The beads were washed four times with cold 1× PBS containing 0.05% (by volume) NP-40 by inverting the tubes 20 times. Post-washing, the beads were resuspended in 0.5 ml of 50 mM tris (pH 7.5), and 100 μl of the bead slurry was used for Western blotting.

For reduction, the bead pellet was resuspended in 50 μl of 5% SDS-TEABC (12.5 μl of 20% SDS and 40 μl of 50 mM TEABC) and 5 mM TCEP [2.5 μl of 100 mM stock prepared in 50 mM TEAB (pH 8.5)] and incubated at 60°C for 30 min at 1200 rpm on a ThermoMixer with lid. After cooling to room temperature, 20 mM IAA (10 μl of 100 mM stock made in milli-Q water) was added and incubated at room temperature for 30 min at 1200 rpm on a ThermoMixer. Alkylation was quenched with 2.5 mM TCEP (1.25 μl per tube) for 10 min at room temperature, and the samples were stored at −80°C. Next day, the samples were thawed, and 6 μl of 27.5% phosphoric acid was added and briefly vortexed. The beads were immobilized, the supernatant was collected in a fresh low binding tube, and the volume was made to 0.5 ml with the binding buffer (5 ml of 1 M TEAB and 45 ml of methanol). Trypsinization and peptide elution were performed using the S-Trap microcolumn as per the manufacturer’s protocol (Protifi, C02-micro-80). Briefly, 20 μg of trypsin/LysC mix (MS grade; Pierce, 1863520) was reconstituted in 200 μl of 100 mM TEAB to a stock solution of 0.1 μg/ml. Twenty microliters of this solution (2 μg) was loaded onto the trap column and incubated at 47°C for 2 hours on a ThermoMixer with lid and no shaking. Digested peptides were sequentially eluted using 40 μl of Elution Buffer 1 [50 mM TEAB in water (pH 8.5)], Elution Buffer 2 (0.2% formic acid in water), and Elution Buffer 3 (50% acetonitrile in water) by centrifugation at 1500*g* for 1 min. The eluted peptides were pooled, dried in a SpeedVac for 2 hours, and resuspended in 20 μl of 5% formic acid in water.

Peptides were analyzed by injecting 200 ng onto a Vanquish Neo UHPLC System operating in trap and elute mode coupled with an Orbitrap Astral Mass Spectrometer (Thermo Fisher Scientific). Peptides were loaded onto a PepMap Neo Trap Cartridge (Thermo Fisher Scientific, no. 174500) and analyzed on a C18 EASY-Spray HPLC Column (Thermo Fisher Scientific, no. ES906) with a 11.8-min gradient from 1 to 55% Buffer B (Buffer A, 0.1% formic acid in water; Buffer B, 0.08% formic acid in 80:20 acetonitrile:water; 0.7 min at 1.8 μl/min from 1 to 4% B, 0.3 min at 1.8 μl/min from 4 to 8% B, 6.7 min at 1.8 μl/min from 8 to 22.5% B, 3.7 min at 1.8 μl/min from 22.5 to 35% B, and 0.4 min at 2.5 μl/min from 35 to 55% B). Eluted peptides were analyzed using data-independent acquisition mode on the mass spectrometer.

### Streptavidin enrichment of biotinylated proteins for Western blot analysis

For Western blot analysis, 2 mg of lysate was incubated with 20 μl of streptavidin magnetic beads for 2 hours at 4°C with gentle rotation. The beads were washed thrice with cold 1× PBS containing 0.05% (by volume) NP-40 and then eluted by boiling the beads for 10 min in 50 μl of 2× NuPAGE LDS buffer containing 2 mM biotin.

### Immunoprecipitation of GFP NRBP1 for MS

To identify the direct interaction with WNK’s and other sorbitol stimulated interactors, GFP-NRBP1 was immunoprecipitated from the GFP-NRBP1 KI HEK293 cells with WT HEK293 serving as negative and bead control. The experiment involved five technical replicates for each of the four groups, namely, GFP-NRBP1 KI HEK293 and WT HEK293 with and without 0.5 M sorbitol for 30 min. Around 4 mg of the lysate in 1 ml of lysis buffer [20 mM Hepes (pH 7.5),150 mM NaCl,1 mM EDTA, 1 mM EGTA, 0.5% (by volume) NP-40, 1 mM NaVO4, 5 mM Na pyrophosphate, 50 mM sodium fluoride, 10 mM β-glycerophosphate, and a protease inhibitor mini tab] was immunoprecipitated using GFP magnetic beads (GFP-Trap Magnetic Particles M-270 Kit). The magnetic bead slurry (25 μl) per IP was used, and the immunoprecipitation was carried out at 4°C for 2 hours. The beads were then immobilized on a magnetic rack, and 50 μl of the supernatant was collected to check for the depletion of GFP-NRBP1.

The beads were washed by inverting the tubes 20 times with Wash Buffer 1 [lysis buffer with 0.1% (by volume) NP-40] twice; Wash Buffer 2 (lysis buffer without NP-40) twice; and Wash Buffer 3 [20 mM Hepes (pH 7.5)] to remove salts. The beads were resuspended in 0.5 ml of 20 mM Hepes, and 10% (50 μl) of the bead slurry was reserved for Western blotting. The reduction, alkylation, trypsinization, and elution steps were the same as that for streptavidin enrichment of biotinylated proteins. Peptides were analyzed by injecting 2 μg onto an UltiMate 3000 RSLCnano System coupled with an Orbitrap Fusion Lumos Tribrid Mass Spectrometer (Thermo Fisher Scientific). Peptides were loaded onto an Acclaim PepMap trap column (Thermo Fisher Scientific, no. 164750) and analyzed on a C18 EASY-Spray HPLC Column (Thermo Fisher Scientific, no. ES903) with a 120-min linear gradient from 3 to 35% Buffer B (Buffer A, 0.1% formic acid in water; Buffer B, 0.08% formic acid in 80:20 acetonitrile:water). Eluted peptides were analyzed using data-independent acquisition mode on the mass spectrometer.

Peptides were searched against the UniProt SwissProt Human database (released on 5 October 2021) using DiaNN (v1.8.1) in library-free mode. Statistical analysis was performed with Python (v3.9.0) and the following packages: pandas (v1.3.3), numpy (v1.19.0), sklearn (v1.0), scipy (v1.7.1), rpy2 (v3.4.5), Plotnine (v0.7.1), and Plotly (v5.8.2). Additionally, R (v4.1.3) and the Limma package (v3.50.1) were used for analysis. Protein groups with fewer than two proteotypic peptides or quantified in fewer than three replicates were excluded from further analysis. Missing values were imputed using a Gaussian distribution centered on the median, with a downshift of 1.8 and a width of 0.3 relative to the standard deviation, and protein intensities were median-normalized. Protein regulation was assessed using LIMMA, with *P* values adjusted by the Benjamini-Hochberg multiple hypothesis correction. Proteins were considered significantly regulated if their corrected *P* value was less than 0.05 and their fold change was either greater than 1.5 or less than 1/1.5.

### AF3 modeling

Modeling of structures of individual proteins and protein complexes was performed using AF3 (*62*) with primary sequences obtained from UniProt: NRBP1 (Q9UHY1), SPAK (Q9UEW8), TSC22D2 (O75157), TSC22D4 (Q9Y3Q8), WNK1 (Q9H4A3), WNK2 (Q9Y3S1), WNK3 (Q9BYP7), and WNK4 (Q96J92). The full-length sequences were used to predict the structures of monomers of NRBP1, WNK1, WNK2, WNK3, WNK4, TSC22D2, and TSC22D4; homo- and heterodimers of TSC22D2 and TSC22D4; homodimer of NRBP1; complexes of NRBP1 with TSC22D2 or TSC22D4; as well as WNK1 in complex with NRBP1, WNK1 in complex with SPAK and NRBP1, and WNK1 in complex with SPAK, NRBP1, and TSC22D4 homodimer. Detailed intermolecular interactions for models of NRBP1 in complex with TSC22D2 or TSC22D4 were analyzed with BIOVIA Discovery Studio Visualizer 2024 using the Nonbonded Interaction Monitor. All structures were visualized using PyMOL 3.0.

## References

[1] B. Xu, J. M. English, J. L. Wilsbacher, S. Stippec, E. J. Goldsmith, M. H. Cobb, WNK1, a novel mammalian serine/threonine protein kinase lacking the catalytic lysine in subdomain II. J. Biol. Chem. 275, 16795–16801 (2000). 10828064 10.1074/jbc.275.22.16795

[2] D. R. Alessi, J. Zhang, A. Khanna, T. Hochdorfer, Y. Shang, K. T. Kahle, The WNK-SPAK/OSR1 pathway: Master regulator of cation-chloride cotransporters. Sci. Signal. 7, re3 (2014). 25028718 10.1126/scisignal.2005365

[3] A. C. Vitari, M. Deak, N. A. Morrice, D. R. Alessi, The WNK1 and WNK4 protein kinases that are mutated in Gordon’s hypertension syndrome phosphorylate and activate SPAK and OSR1 protein kinases. Biochem. J. 391, 17–24 (2005). 16083423 10.1042/BJ20051180 PMC1237134

[4] A. C. Vitari, J. Thastrup, F. H. Rafiqi, M. Deak, N. A. Morrice, H. K. Karlsson, D. R. Alessi, Functional interactions of the SPAK/OSR1 kinases with their upstream activator WNK1 and downstream substrate NKCC1. Biochem. J. 397, 223–231 (2006). 16669787 10.1042/BJ20060220 PMC1479760

[5] B. E. Xu, X. Min, S. Stippec, B. H. Lee, E. J. Goldsmith, M. H. Cobb, Regulation of WNK1 by an autoinhibitory domain and autophosphorylation. J. Biol. Chem. 277, 48456–48462 (2002). 12374799 10.1074/jbc.M207917200

[6] J. Zhang, K. Siew, T. Macartney, K. M. O’Shaughnessy, D. R. Alessi, Critical role of the SPAK protein kinase CCT domain in controlling blood pressure. Hum. Mol. Genet. 24, 4545–4558 (2015). 25994507 10.1093/hmg/ddv185 PMC4512625

[7] F. Villa, J. Goebel, F. H. Rafiqi, M. Deak, J. Thastrup, D. R. Alessi, D. M. van Aalten, Structural insights into the recognition of substrates and activators by the OSR1 kinase. EMBO Rep. 8, 839–845 (2007). 17721439 10.1038/sj.embor.7401048 PMC1973955

[8] K. B. Gagnon, R. England, E. Delpire, A single binding motif is required for SPAK activation of the Na-K-2Cl cotransporter. Cell. Physiol. Biochem. 20, 131–142 (2006). 10.1159/000104161 17595523

[9] S. G. Bartual, D. M. Pinkas, J. C. Bufton, K. Kupinska, D. Wang, R. Chalk, G. Berridge, N. A. Burgess-Brown, F. von Delft, C. H. Arrowsmith, A. M. Edwards, C. Bountra, A. Bullock, Structural Genomics Consortium (SGC), Crystal structure of WNK3 kinase and CCT1 didomain in a unphosphorylated state, Protein Data Bank (2017); 10.2210/pdb5O2C/pdb.

[10] T. M. Moon, F. Correa, L. N. Kinch, A. T. Piala, K. H. Gardner, E. J. Goldsmith, Solution structure of the WNK1 autoinhibitory domain, a WNK-specific PF2 domain. J. Mol. Biol. 425, 1245–1252 (2013). 23376100 10.1016/j.jmb.2013.01.031

[11] D. M. Pinkas, J. C. Bufton, N. A. Burgess-Brown, C. H. Arrowsmith, A. M. Edwards, C. Bountra, A. N. Bullock, Crystal structure of the human WNK2 CCT-like 1 domain in complex with a WNK1 RFXV peptide (6FBK), Protein Data Bank (2017); 10.2210/pdb6fbk/pdb.

[12] C. A. Taylor IV, M. H. Cobb, CCT and CCT-like modular protein interaction domains in WNK signaling. Mol. Pharmacol. 101, 201–212 (2022). 34312216 10.1124/molpharm.121.000307 PMC9092477

[13] S. Uchida, T. Mori, K. Susa, E. Sohara, NCC regulation by WNK signal cascade. Front. Physiol. 13, 1081261 (2022). 36685207 10.3389/fphys.2022.1081261 PMC9845728

[14] R. Akella, J. M. Humphreys, K. Sekulski, H. He, M. Durbacz, S. Chakravarthy, J. Liwocha, Z. J. Mohammed, C. A. Brautigam, E. J. Goldsmith, Osmosensing by WNK kinases. Mol. Biol. Cell 32, 1614–1623 (2021). 33689398 10.1091/mbc.E20-01-0089 PMC8684725

[15] A. R. Murillo-de-Ozores, M. Chavez-Canales, P. de Los Heros, G. Gamba, M. Castaneda-Bueno, Physiological processes modulated by the chloride-sensitive WNK-SPAK/OSR1 kinase signaling pathway and the cation-coupled chloride cotransporters. Front. Physiol. 11, 585907 (2020). 33192599 10.3389/fphys.2020.585907 PMC7606576

[16] P. de Los Heros, D. Pacheco-Alvarez, G. Gamba, Role of WNK kinases in the modulation of cell volume. Curr. Top. Membr. 81, 207–235 (2018). 30243433 10.1016/bs.ctm.2018.08.002

[17] F. H. Rafiqi, A. M. Zuber, M. Glover, C. Richardson, S. Fleming, S. Jovanovic, A. Jovanovic, K. M. O’Shaughnessy, D. R. Alessi, Role of the WNK-activated SPAK kinase in regulating blood pressure. EMBO Mol. Med. 2, 63–75 (2010). 20091762 10.1002/emmm.200900058 PMC3377268

[18] C. Richardson, K. Sakamoto, P. de los Heros, M. Deak, D. G. Campbell, A. R. Prescott, D. R. Alessi, Regulation of the NKCC2 ion cotransporter by SPAK-OSR1-dependent and -independent pathways. J. Cell Sci. 124, 789–800 (2011). 21321328 10.1242/jcs.077230 PMC3114804

[19] K. B. Gagnon, E. Delpire, On the substrate recognition and negative regulation of SPAK, a kinase modulating Na^+^-K^+^-2Cl^-^ cotransport activity. Am. J. Physiol. Cell Physiol. 299, C614–C620 (2010). 20463172 10.1152/ajpcell.00074.2010 PMC2944316

[20] D. Pacheco-Alvarez, P. S. Cristobal, P. Meade, E. Moreno, N. Vazquez, E. Munoz, A. Diaz, M. E. Juarez, I. Gimenez, G. Gamba, The Na^+^:Cl- cotransporter is activated and phosphorylated at the amino-terminal domain upon intracellular chloride depletion. J. Biol. Chem. 281, 28755–28763 (2006). 16887815 10.1074/jbc.M603773200

[21] J. Zhang, G. Gao, G. Begum, J. Wang, A. R. Khanna, B. E. Shmukler, G. M. Daubner, P. de Los Heros, P. Davies, J. Varghese, M. I. Bhuiyan, J. Duan, J. Zhang, D. Duran, S. L. Alper, D. Sun, S. J. Elledge, D. R. Alessi, K. T. Kahle, Functional kinomics establishes a critical node of volume-sensitive cation-Cl^−^ cotransporter regulation in the mammalian brain. Sci. Rep. 6, 35986 (2016). 27782176 10.1038/srep35986 PMC5080614

[22] N. C. Adragna, N. B. Ravilla, P. K. Lauf, G. Begum, A. R. Khanna, D. Sun, K. T. Kahle, Regulated phosphorylation of the K-Cl cotransporter KCC3 is a molecular switch of intracellular potassium content and cell volume homeostasis. Front. Cell. Neurosci. 9, 255 (2015). 26217182 10.3389/fncel.2015.00255 PMC4496573

[23] P. de Los Heros, D. R. Alessi, R. Gourlay, D. G. Campbell, M. Deak, T. J. Macartney, K. T. Kahle, J. Zhang, The WNK-regulated SPAK/OSR1 kinases directly phosphorylate and inhibit the K^+^-Cl^−^ co-transporters. Biochem. J. 458, 559–573 (2014). 24393035 10.1042/BJ20131478 PMC3940040

[24] J. Rinehart, Y. D. Maksimova, J. E. Tanis, K. L. Stone, C. A. Hodson, J. Zhang, M. Risinger, W. Pan, D. Wu, C. M. Colangelo, B. Forbush, C. H. Joiner, E. E. Gulcicek, P. G. Gallagher, R. P. Lifton, Sites of regulated phosphorylation that control K-Cl cotransporter activity. Cell 138, 525–536 (2009). 19665974 10.1016/j.cell.2009.05.031 PMC2811214

[25] S. S. Josiah, N. F. Meor Azlan, J. Zhang, Targeting the WNK-SPAK/OSR1 pathway and cation-chloride cotransporters for the therapy of stroke. Int. J. Mol. Sci. 22, 1232 (2021). 33513812 10.3390/ijms22031232 PMC7865768

[26] M. Chavez-Canales, C. Zhang, C. Soukaseum, E. Moreno, D. Pacheco-Alvarez, E. Vidal-Petiot, M. Castaneda-Bueno, N. Vazquez, L. Rojas-Vega, N. P. Meermeier, S. Rogers, X. Jeunemaitre, C. L. Yang, D. H. Ellison, G. Gamba, J. Hadchouel, WNK-SPAK-NCC cascade revisited: WNK1 stimulates the activity of the Na-Cl cotransporter via SPAK, an effect antagonized by WNK4. Hypertension 64, 1047–1053 (2014). 25113964 10.1161/HYPERTENSIONAHA.114.04036 PMC5832045

[27] E. J. Hoorn, J. H. Nelson, J. A. McCormick, D. H. Ellison, The WNK kinase network regulating sodium, potassium, and blood pressure. J. Am. Soc. Nephrol. 22, 605–614 (2011). 21436285 10.1681/ASN.2010080827 PMC4496838

[28] P. San-Cristobal, P. de los Heros, J. Ponce-Coria, E. Moreno, G. Gamba, WNK kinases, renal ion transport and hypertension. Am. J. Nephrol. 28, 860–870 (2008). 18547946 10.1159/000139639 PMC2820349

[29] F. H. Wilson, S. Disse-Nicodeme, K. A. Choate, K. Ishikawa, C. Nelson-Williams, I. Desitter, M. Gunel, D. V. Milford, G. W. Lipkin, J. M. Achard, M. P. Feely, B. Dussol, Y. Berland, R. J. Unwin, H. Mayan, D. B. Simon, Z. Farfel, X. Jeunemaitre, R. P. Lifton, Human hypertension caused by mutations in WNK kinases. Science 293, 1107–1112 (2001). 11498583 10.1126/science.1062844

[30] S. Gallolu Kankanamalage, A. Y. Lee, C. Wichaidit, A. Lorente-Rodriguez, A. M. Shah, S. Stippec, A. W. Whitehurst, M. H. Cobb, WNK1 is an unexpected autophagy inhibitor. Autophagy 13, 969–970 (2017). 28282258 10.1080/15548627.2017.1286431 PMC5446055

[31] M. Shekarabi, J. Zhang, A. R. Khanna, D. H. Ellison, E. Delpire, K. T. Kahle, WNK kinase signaling in ion homeostasis and human disease. Cell Metab. 25, 285–299 (2017). 28178566 10.1016/j.cmet.2017.01.007

[32] A. B. Jaykumar, J. U. Jung, P. K. Parida, T. T. Dang, C. Wichaidit, A. R. Kannangara, S. Earnest, E. J. Goldsmith, G. W. Pearson, S. Malladi, M. H. Cobb, WNK1 enhances migration and invasion in breast cancer models. Mol. Cancer Ther. 20, 1800–1808 (2021). 34253593 10.1158/1535-7163.MCT-21-0174 PMC9013269

[33] A. Sato, H. Shibuya, WNK signaling is involved in neural development via Lhx8/Awh expression. PLOS ONE 8, e55301 (2013). 23383144 10.1371/journal.pone.0055301 PMC3559379

[34] S. W. Tu, A. Bugde, K. Luby-Phelps, M. H. Cobb, WNK1 is required for mitosis and abscission. Proc. Natl. Acad. Sci. U.S.A. 108, 1385–1390 (2011). 21220314 10.1073/pnas.1018567108 PMC3029763

[35] R. Kochl, F. Thelen, L. Vanes, T. F. Brazao, K. Fountain, J. Xie, C. L. Huang, R. Lyck, J. V. Stein, V. L. Tybulewicz, WNK1 kinase balances T cell adhesion versus migration in vivo. Nat. Immunol. 17, 1075–1083 (2016). 27400149 10.1038/ni.3495 PMC4994873

[36] J. Biggs O’May, L. Vanes, L. L. de Boer, D. A. Lewis, H. Hartweger, S. Kunzelmann, D. Hayward, M. Llorian, R. Kochl, V. L. J. Tybulewicz, WNK1-dependent water influx is required for CD4^+^ T cell activation and T cell-dependent antibody responses. Nat. Commun. 16, 1857 (2025). 39984435 10.1038/s41467-025-56778-x PMC11845700

[37] G. Jiang, Y. Cai, D. Cheng, H. Wang, G. Deng, D. Xiang, CYLD alleviates NLRP3 inflammasome-mediated pyroptosis in osteoporosis by deubiquitinating WNK1. J. Orthop. Surg. Res. 19, 212 (2024). 38561786 10.1186/s13018-024-04675-2 PMC10983667

[38] L. Mayes-Hopfinger, A. Enache, J. Xie, C. L. Huang, R. Kochl, V. L. J. Tybulewicz, T. Fernandes-Alnemri, E. S. Alnemri, Chloride sensing by WNK1 regulates NLRP3 inflammasome activation and pyroptosis. Nat. Commun. 12, 4546 (2021). 34315884 10.1038/s41467-021-24784-4 PMC8316491

[39] C. R. Boyd-Shiwarski, D. J. Shiwarski, S. E. Griffiths, R. T. Beacham, L. Norrell, D. E. Morrison, J. Wang, J. Mann, W. Tennant, E. N. Anderson, J. Franks, M. Calderon, K. A. Connolly, M. U. Cheema, C. J. Weaver, L. J. Nkashama, C. C. Weckerly, K. E. Querry, U. B. Pandey, C. J. Donnelly, D. Sun, A. R. Rodan, A. R. Subramanya, WNK kinases sense molecular crowding and rescue cell volume via phase separation. Cell 185, 4488–4506.e20 (2022). 36318922 10.1016/j.cell.2022.09.042 PMC9699283

[40] J. O. Thastrup, F. H. Rafiqi, A. C. Vitari, E. Pozo-Guisado, M. Deak, Y. Mehellou, D. R. Alessi, SPAK/OSR1 regulate NKCC1 and WNK activity: Analysis of WNK isoform interactions and activation by T-loop trans-autophosphorylation. Biochem. J. 441, 325–337 (2012). 22032326 10.1042/BJ20111879 PMC3242505

[41] A. Ohta, F. R. Schumacher, Y. Mehellou, C. Johnson, A. Knebel, T. J. Macartney, N. T. Wood, D. R. Alessi, T. Kurz, The CUL3-KLHL3 E3 ligase complex mutated in Gordon’s hypertension syndrome interacts with and ubiquitylates WNK isoforms: Disease-causing mutations in KLHL3 and WNK4 disrupt interaction. Biochem. J. 451, 111–122 (2013). 23387299 10.1042/BJ20121903 PMC3632089

[42] T. Pleiner, M. Hazu, G. P. Tomaleri, K. Januszyk, R. S. Oania, M. J. Sweredoski, A. Moradian, A. Guna, R. M. Voorhees, WNK1 is an assembly factor for the human ER membrane protein complex. Mol. Cell 81, 2693–2704.e12 (2021). 33964204 10.1016/j.molcel.2021.04.013 PMC8254792

[43] Y. Arai, K. Asano, S. Mandai, F. Ando, K. Susa, T. Mori, N. Nomura, T. Rai, M. Tanaka, S. Uchida, E. Sohara, WNK1-TAK1 signaling suppresses lipopolysaccharide-induced cytokine production and classical activation in macrophages. Biochem. Biophys. Res. Commun. 533, 1290–1297 (2020). 33046244 10.1016/j.bbrc.2020.10.007

[44] Z. Zhang, X. Xu, Y. Zhang, J. Zhou, Z. Yu, C. He, LINGO-1 interacts with WNK1 to regulate nogo-induced inhibition of neurite extension. J. Biol. Chem. 284, 15717–15728 (2009). 19363035 10.1074/jbc.M808751200 PMC2708869

[45] B. E. Xu, S. Stippec, P. Y. Chu, A. Lazrak, X. J. Li, B. H. Lee, J. M. English, B. Ortega, C. L. Huang, M. H. Cobb, WNK1 activates SGK1 to regulate the epithelial sodium channel. Proc. Natl. Acad. Sci. U.S.A. 102, 10315–10320 (2005). 16006511 10.1073/pnas.0504422102 PMC1177404

[46] W. Qin, K. F. Cho, P. E. Cavanagh, A. Y. Ting, Deciphering molecular interactions by proximity labeling. Nat. Methods 18, 133–143 (2021). 33432242 10.1038/s41592-020-01010-5 PMC10548357

[47] T. C. Branon, J. A. Bosch, A. D. Sanchez, N. D. Udeshi, T. Svinkina, S. A. Carr, J. L. Feldman, N. Perrimon, A. Y. Ting, Efficient proximity labeling in living cells and organisms with TurboID. Nat. Biotechnol. 36, 880–887 (2018). 30125270 10.1038/nbt.4201 PMC6126969

[48] S. Roth, N. M. Kocaturk, P. S. Sathyamurthi, B. Carton, M. Watt, T. J. Macartney, K. H. Chan, A. Isidro-Llobet, A. Konopacka, M. A. Queisser, G. P. Sapkota, Identification of KLHDC2 as an efficient proximity-induced degrader of K-RAS, STK33, β-catenin, and FoxP3. Cell Chem. Biol. 30, 1261–1276.e7 (2023). 37591251 10.1016/j.chembiol.2023.07.006

[49] S. Gluderer, E. Brunner, M. Germann, V. Jovaisaite, C. Li, C. A. Rentsch, E. Hafen, H. Stocker, Madm (Mlf1 adapter molecule) cooperates with Bunched A to promote growth in *Drosophila*. J. Biol. 9, 9 (2010). 20149264 10.1186/jbiol216 PMC2871527

[50] C. H. Wilson, C. Crombie, L. van der Weyden, G. Poulogiannis, A. G. Rust, M. Pardo, T. Gracia, L. Yu, J. Choudhary, G. B. Poulin, R. E. McIntyre, D. J. Winton, H. N. March, M. J. Arends, A. G. Fraser, D. J. Adams, Nuclear receptor binding protein 1 regulates intestinal progenitor cell homeostasis and tumour formation. EMBO J. 31, 2486–2497 (2012). 22510880 10.1038/emboj.2012.91 PMC3365428

[51] T. Yasukawa, A. Tsutsui, C. Tomomori-Sato, S. Sato, A. Saraf, M. P. Washburn, L. Florens, T. Terada, K. Shimizu, R. C. Conaway, J. W. Conaway, T. Aso, NRBP1-containing CRL2/CRL4A regulates amyloid β production by targeting BRI2 and BRI3 for degradation. Cell Rep. 30, 3478–3491.e6 (2020). 32160551 10.1016/j.celrep.2020.02.059

[52] Y. X. Xiao, S. Y. Lee, M. Aguilera-Uribe, R. Samson, A. Au, Y. Khanna, Z. Liu, R. Cheng, K. Aulakh, J. Wei, A. G. Farias, T. Reilly, S. Birkadze, A. Habsid, K. R. Brown, K. Chan, P. Mero, J. Q. Huang, M. Billmann, M. Rahman, C. Myers, B. J. Andrews, J. Y. Youn, C. M. Yip, D. Rotin, W. B. Derry, J. D. Forman-Kay, A. M. Moses, I. Pritisanac, A. C. Gingras, J. Moffat, The TSC22D, WNK, and NRBP gene families exhibit functional buffering and evolved with Metazoa for cell volume regulation. Cell Rep. 43, 114417 (2024). 38980795 10.1016/j.celrep.2024.114417

[53] G. R. Magana-Avila, H. Carbajal-Contreras, R. Amnekar, T. Dite, M. Tellez-Sutterlin, K. Garcia-Avila, B. Marquina-Castillo, A. Lopez-Saavedra, N. Vazquez, E. Rojas-Ortega, E. Delpire, D. H. Ellison, D. R. Alessi, G. Gamba, M. Castaneda-Bueno, NRBP1 and TSC22D proteins impact distal convoluted tubule physiology through modulation of the WNK pathway. bioRxiv 628222 [Preprint] (2024). 10.1101/2024.12.12.628222.

[54] J. Boudeau, D. Miranda-Saavedra, G. J. Barton, D. R. Alessi, Emerging roles of pseudokinases. Trends Cell Biol. 16, 443–452 (2006). 16879967 10.1016/j.tcb.2006.07.003

[55] J. S. Kerr, C. H. Wilson, Nuclear receptor-binding protein 1: A novel tumour suppressor and pseudokinase. Biochem. Soc. Trans. 41, 1055–1060 (2013). 23863178 10.1042/BST20130069

[56] G. Manning, D. B. Whyte, R. Martinez, T. Hunter, S. Sudarsanam, The protein kinase complement of the human genome. Science 298, 1912–1934 (2002). 12471243 10.1126/science.1075762

[57] C. A. Taylor, J.-U. Jung, S. G. Kankanamalage, J. Li, M. Grzemska, A. B. Jaykumar, S. Earnest, S. Stippec, P. Saha, E. Sauceda, M. H. Cobb, Predictive and experimental motif interaction analysis identifies functions of the WNK-OSR1/SPAK Pathway. bioRxiv 600905 [Preprint] (2024). 10.1101/2024.06.26.600905.

[58] D. F. Fiol, S. K. Mak, D. Kultz, Specific TSC22 domain transcripts are hypertonically induced and alternatively spliced to protect mouse kidney cells during osmotic stress. FEBS J. 274, 109–124 (2007). 17147695 10.1111/j.1742-4658.2006.05569.x

[59] J. M. McFarland, Z. V. Ho, G. Kugener, J. M. Dempster, P. G. Montgomery, J. G. Bryan, J. M. Krill-Burger, T. M. Green, F. Vazquez, J. S. Boehm, T. R. Golub, W. C. Hahn, D. E. Root, A. Tsherniak, Improved estimation of cancer dependencies from large-scale RNAi screens using model-based normalization and data integration. Nat. Commun. 9, 4610 (2018). 30389920 10.1038/s41467-018-06916-5 PMC6214982

[60] R. M. Meyers, J. G. Bryan, J. M. McFarland, B. A. Weir, A. E. Sizemore, H. Xu, N. V. Dharia, P. G. Montgomery, G. S. Cowley, S. Pantel, A. Goodale, Y. Lee, L. D. Ali, G. Jiang, R. Lubonja, W. F. Harrington, M. Strickland, T. Wu, D. C. Hawes, V. A. Zhivich, M. R. Wyatt, Z. Kalani, J. J. Chang, M. Okamoto, K. Stegmaier, T. R. Golub, J. S. Boehm, F. Vazquez, D. E. Root, W. C. Hahn, A. Tsherniak, Computational correction of copy number effect improves specificity of CRISPR-Cas9 essentiality screens in cancer cells. Nat. Genet. 49, 1779–1784 (2017). 29083409 10.1038/ng.3984 PMC5709193

[61] J. D. Hooper, E. Baker, S. M. Ogbourne, G. R. Sutherland, T. M. Antalis, Cloning of the cDNA and localization of the gene encoding human NRBP, a ubiquitously expressed, multidomain putative adapter protein. Genomics 66, 113–118 (2000). 10843813 10.1006/geno.2000.6167

[62] J. Abramson, J. Adler, J. Dunger, R. Evans, T. Green, A. Pritzel, O. Ronneberger, L. Willmore, A. J. Ballard, J. Bambrick, S. W. Bodenstein, D. A. Evans, C. C. Hung, M. O’Neill, D. Reiman, K. Tunyasuvunakool, Z. Wu, A. Zemgulyte, E. Arvaniti, C. Beattie, O. Bertolli, A. Bridgland, A. Cherepanov, M. Congreve, A. I. Cowen-Rivers, A. Cowie, M. Figurnov, F. B. Fuchs, H. Gladman, R. Jain, Y. A. Khan, C. M. R. Low, K. Perlin, A. Potapenko, P. Savy, S. Singh, A. Stecula, A. Thillaisundaram, C. Tong, S. Yakneen, E. D. Zhong, M. Zielinski, A. Zidek, V. Bapst, P. Kohli, M. Jaderberg, D. Hassabis, J. M. Jumper, Accurate structure prediction of biomolecular interactions with AlphaFold 3. Nature 630, 493–500 (2024). 38718835 10.1038/s41586-024-07487-w PMC11168924

[63] H. A. Kester, C. Blanchetot, J. den Hertog, P. T. van der Saag, B. van der Burg, Transforming growth factor-β-stimulated clone-22 is a member of a family of leucine zipper proteins that can homo- and heterodimerize and has transcriptional repressor activity. J. Biol. Chem. 274, 27439–27447 (1999). 10488076 10.1074/jbc.274.39.27439

[64] F. Liang, Q. Li, X. Li, Z. Li, Z. Gong, H. Deng, B. Xiang, M. Zhou, X. Li, G. Li, Z. Zeng, W. Xiong, TSC22D2 interacts with PKM2 and inhibits cell growth in colorectal cancer. Int. J. Oncol. 49, 1046–1056 (2016). 27573352 10.3892/ijo.2016.3599

[65] R. Evans, M. O’Neill, A. Pritzel, N. Antropova, A. Senior, T. Green, A. Žídek, R. Bates, S. Blackwell, J. Yim, O. Ronneberger, S. Bodenstein, M. Zielinski, A. Bridgland, A. Potapenko, A. Cowie, K. Tunyasuvunakool, R. Jain, E. Clancy, P. Kohli, J. Jumper, D. Hassabis, Protein complex prediction with AlphaFold-Multimer. bioRxiv 463034 [Preprint] (2022). 10.1101/2021.10.04.463034.

[66] K. Yamada, H. M. Park, D. F. Rigel, K. DiPetrillo, E. J. Whalen, A. Anisowicz, M. Beil, J. Berstler, C. E. Brocklehurst, D. A. Burdick, S. L. Caplan, M. P. Capparelli, G. Chen, W. Chen, B. Dale, L. Deng, F. Fu, N. Hamamatsu, K. Harasaki, T. Herr, P. Hoffmann, Q. Y. Hu, W. J. Huang, N. Idamakanti, H. Imase, Y. Iwaki, M. Jain, J. Jeyaseelan, M. Kato, V. K. Kaushik, D. Kohls, V. Kunjathoor, D. LaSala, J. Lee, J. Liu, Y. Luo, F. Ma, R. Mo, S. Mowbray, M. Mogi, F. Ossola, P. Pandey, S. J. Patel, S. Raghavan, B. Salem, Y. H. Shanado, G. M. Trakshel, G. Turner, H. Wakai, C. Wang, S. Weldon, J. B. Wielicki, X. Xie, L. Xu, Y. I. Yagi, K. Yasoshima, J. Yin, D. Yowe, J. H. Zhang, G. Zheng, L. Monovich, Small-molecule WNK inhibition regulates cardiovascular and renal function. Nat. Chem. Biol. 12, 896–898 (2016). 27595330 10.1038/nchembio.2168

[67] V. Demichev, C. B. Messner, S. I. Vernardis, K. S. Lilley, M. Ralser, DIA-NN: Neural networks and interference correction enable deep proteome coverage in high throughput. Nat. Methods 17, 41–44 (2020). 31768060 10.1038/s41592-019-0638-x PMC6949130

[68] T. K. Phung, K. Berndsen, R. Shastry, T. Phan, M. M. K. Muqit, D. R. Alessi, R. S. Nirujogi, CURTAIN-A unique web-based tool for exploration and sharing of MS-based proteomics data. Proc. Natl. Acad. Sci. U.S.A. 121, e2312676121 (2024). 38324566 10.1073/pnas.2312676121 PMC10873628

[69] A. G. Bond, C. Craigon, K. H. Chan, A. Testa, A. Karapetsas, R. Fasimoye, T. Macartney, J. J. Blow, D. R. Alessi, A. Ciulli, Development of BromoTag: A “Bump-and-Hole”-PROTAC system to induce potent, rapid, and selective degradation of tagged target proteins. J. Med. Chem. 64, 15477–15502 (2021). 34652918 10.1021/acs.jmedchem.1c01532 PMC8558867

[70] S. De Langhe, L. Haataja, D. Senadheera, J. Groffen, N. Heisterkamp, Interaction of the small GTPase Rac3 with NRBP, a protein with a kinase-homology domain. Int. J. Mol. Med. 9, 451–459 (2002). 11956649

[71] M. B. Elkins, J. J. Henry, Isolation and characterization of a novel gene, xMADML, involved in Xenopus laevis eye development. Dev. Dyn. 235, 1845–1857 (2006). 16607642 10.1002/dvdy.20824

[72] H. Wang, X. Sun, Y. Luo, Z. Lin, J. Wu, Adapter protein NRBP associates with Jab1 and negatively regulates AP-1 activity. FEBS Lett. 580, 6015–6021 (2006). 17052710 10.1016/j.febslet.2006.10.002

[73] F. Okumura, M. Matsuzaki, K. Nakatsukasa, T. Kamura, The role of elongin BC-containing ubiquitin ligases. Front. Oncol. 2, 10 (2012). 22649776 10.3389/fonc.2012.00010 PMC3355856

[74] J. M. Murphy, Q. Zhang, S. N. Young, M. L. Reese, F. P. Bailey, P. A. Eyers, D. Ungureanu, H. Hammaren, O. Silvennoinen, L. N. Varghese, K. Chen, A. Tripaydonis, N. Jura, K. Fukuda, J. Qin, Z. Nimchuk, M. B. Mudgett, S. Elowe, C. L. Gee, L. Liu, R. J. Daly, G. Manning, J. J. Babon, I. S. Lucet, A robust methodology to subclassify pseudokinases based on their nucleotide-binding properties. Biochem. J. 457, 323–334 (2014). 24107129 10.1042/BJ20131174 PMC5679212

[75] A. F. Baas, J. Boudeau, G. P. Sapkota, L. Smit, R. Medema, N. A. Morrice, D. R. Alessi, H. C. Clevers, Activation of the tumour suppressor kinase LKB1 by the STE20-like pseudokinase STRAD. EMBO J. 22, 3062–3072 (2003). 12805220 10.1093/emboj/cdg292 PMC162144

[76] J. Boudeau, A. F. Baas, M. Deak, N. A. Morrice, A. Kieloch, M. Schutkowski, A. R. Prescott, H. C. Clevers, D. R. Alessi, MO25α/β interact with STRADα/β enhancing their ability to bind, activate and localize LKB1 in the cytoplasm. EMBO J. 22, 5102–5114 (2003). 14517248 10.1093/emboj/cdg490 PMC204473

[77] F. Shi, S. E. Telesco, Y. Liu, R. Radhakrishnan, M. A. Lemmon, ErbB3/HER3 intracellular domain is competent to bind ATP and catalyze autophosphorylation. Proc. Natl. Acad. Sci. U.S.A. 107, 7692–7697 (2010). 20351256 10.1073/pnas.1002753107 PMC2867849

[78] P. Saharinen, K. Takaluoma, O. Silvennoinen, Regulation of the Jak2 tyrosine kinase by its pseudokinase domain. Mol. Cell. Biol. 20, 3387–3395 (2000). 10779328 10.1128/mcb.20.10.3387-3395.2000 PMC85631

[79] J. Hu, H. Yu, A. P. Kornev, J. Zhao, E. L. Filbert, S. S. Taylor, A. S. Shaw, Mutation that blocks ATP binding creates a pseudokinase stabilizing the scaffolding function of kinase suppressor of Ras, CRAF and BRAF. Proc. Natl. Acad. Sci. U.S.A. 108, 6067–6072 (2011). 21441104 10.1073/pnas.1102554108 PMC3076888

[80] A. T. Piala, T. M. Moon, R. Akella, H. He, M. H. Cobb, E. J. Goldsmith, Chloride sensing by WNK1 involves inhibition of autophosphorylation. Sci. Signal. 7, ra41 (2014). 24803536 10.1126/scisignal.2005050 PMC4123527

[81] A. Zagorska, E. Pozo-Guisado, J. Boudeau, A. C. Vitari, F. H. Rafiqi, J. Thastrup, M. Deak, D. G. Campbell, N. A. Morrice, A. R. Prescott, D. R. Alessi, Regulation of activity and localization of the WNK1 protein kinase by hyperosmotic stress. J. Cell Biol. 176, 89–100 (2007). 17190791 10.1083/jcb.200605093 PMC2063630

[82] T. Tachie-Menson, A. Gazquez-Gutierrez, L. J. Fulcher, T. J. Macartney, N. T. Wood, J. Varghese, R. Gourlay, R. F. Soares, G. P. Sapkota, Characterisation of the biochemical and cellular roles of native and pathogenic amelogenesis imperfecta mutants of FAM83H. Cell. Signal. 72, 109632 (2020). 32289446 10.1016/j.cellsig.2020.109632 PMC7284315

[83] B. Yariv, E. Yariv, A. Kessel, G. Masrati, A. B. Chorin, E. Martz, I. Mayrose, T. Pupko, N. Ben-Tal, Using evolutionary data to make sense of macromolecules with a “face-lifted” ConSurf. Protein Sci. 32, e4582 (2023). 36718848 10.1002/pro.4582 PMC9942591

[84] R. C. Edgar, MUSCLE: Multiple sequence alignment with high accuracy and high throughput. Nucleic Acids Res. 32, 1792–1797 (2004). 15034147 10.1093/nar/gkh340 PMC390337

[85] A. M. Waterhouse, J. B. Procter, D. M. Martin, M. Clamp, G. J. Barton, Jalview Version 2—A multiple sequence alignment editor and analysis workbench. Bioinformatics 25, 1189–1191 (2009). 19151095 10.1093/bioinformatics/btp033 PMC2672624

