# Supplementary material for: NRBP1 pseudokinase binds to and activates the WNK pathway in response to osmotic stress
Source: Sci Adv. 2025 Jul 16;11(29):eadv4636. doi: 10.1126/sciadv.adv4636 (PMC12266122; doi:10.1126/sciadv.adv4636)
Supplement: Supplementary file 1 — Figs. S1 to S9 [file sciadv.adv4636_sm.pdf]

Science Advances
AAAS

Supplementary Materials for

NRBP1 pseudokinase binds to and activates the WNK pathway in response to osmotic stress

Ramchandra V. Amnekar *et al.*

Corresponding author: Ramchandra V. Amnekar, ramnekar001@dundee.ac.uk;
Dario R. Alessi, d.r.alessi@dundee.ac.uk

Sci. Adv. **11**, eadv4636 (2025)
DOI: 10.1126/sciadv.adv4636

This PDF file includes:

Figs. S1 to S9

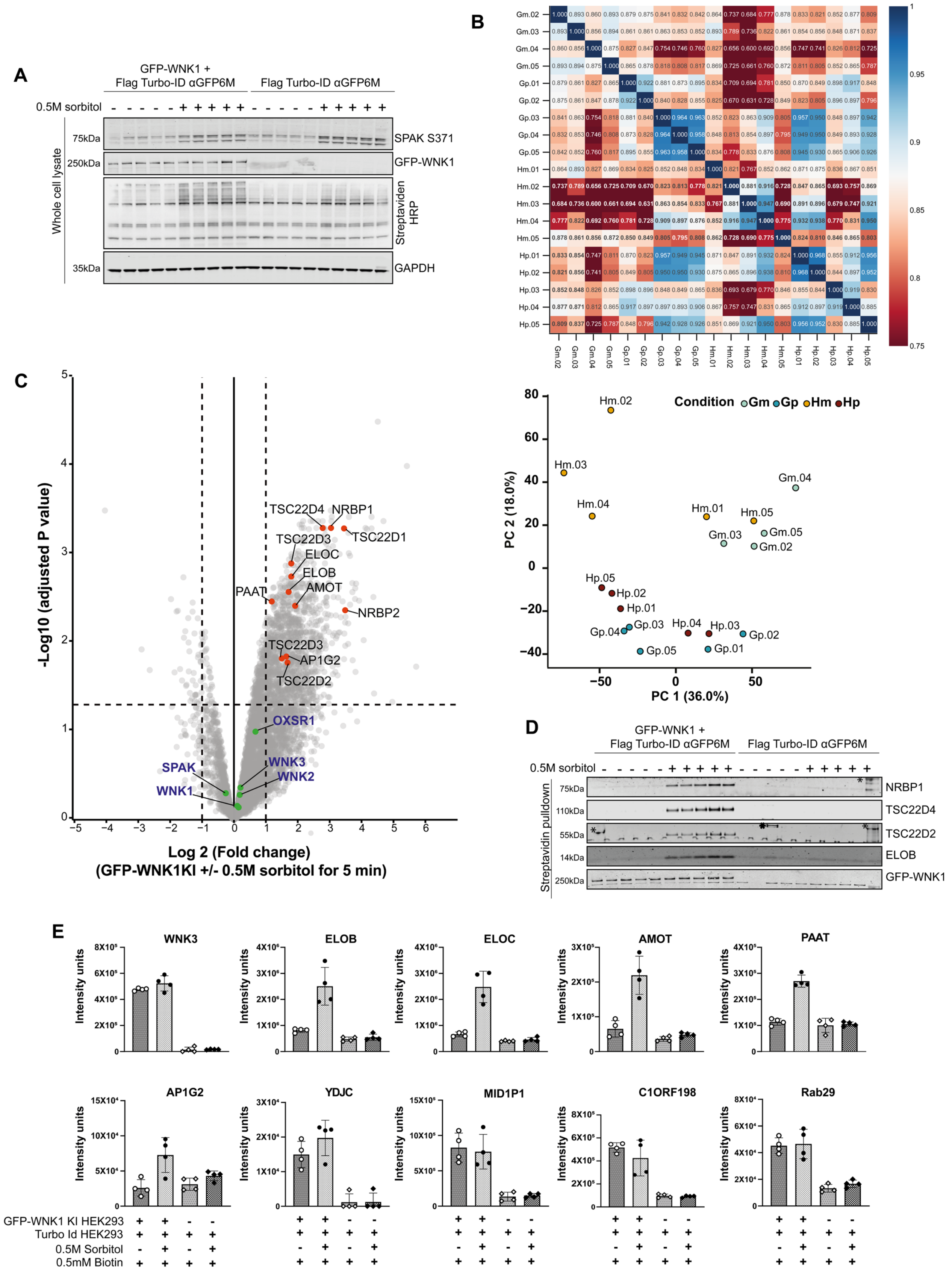

A
GFP-WNK1 +
Flag Turbo-ID αGFP6M
Flag Turbo-ID αGFP6M
0.5M sorbitol
Whole cell lysate
75kDa
250kDa
35kDa
SPAK S371
GFP-WNK1
Streptaviden HRP
GAPDH
B
Gm.02
Gm.03
Gm.04
Gm.05
Gp.01
Gp.02
Gp.03
Gp.04
Gp.05
Hm.01
Hm.02
Hm.03
Hm.04
Hm.05
Hp.01
Hp.02
Hp.03
Hp.04
Hp.05
1
0.95
0.9
0.85
0.8
0.75
C
-Log10 (adjusted P value)
Log 2 (Fold change)
(GFP-WNK1KI +/- 0.5M sorbitol for 5 min)
TSC22D4
NRBP1
TSC22D1
TSC22D3
ELOC
ELOB
PAAT
AMOT
NRBP2
TSC22D3
AP1G2
TSC22D2
OXSR1
SPAK
WNK3
WNK2
WNK1
Condition
Gm
Gp
Hm
Hp
PC 2 (18.0%)
PC 1 (36.0%)
D
GFP-WNK1 +
Flag Turbo-ID αGFP6M
Flag Turbo-ID αGFP6M
0.5M sorbitol
Streptavidin pulldown
75kDa
110kDa
55kDa
14kDa
250kDa
NRBP1
TSC22D4
TSC22D2
ELOB
GFP-WNK1
E
WNK3
ELOB
ELOC
AMOT
PAAT
AP1G2
YDJC
MID1P1
C1ORF198
Rab29
Intensity units
GFP-WNK1 KI HEK293
Turbo Id HEK293
0.5M Sorbitol
0.5mM Biotin

Fig. S1. Analysis of WNK Pathway Activation and Protein Biotinylation Upon Hypertonic Stress (A) Representative immunoblots showing the activation of the WNK signaling pathway (pSPAK blot) and enhanced protein biotinylation (streptavidin-HRP blot) in response to 0.5 M sorbitol treatment for 5 minutes in the presence of 0.5 mM biotin. Data represent biological replicates (n = 5 for each treatment group). **(B)** Top Panel: Heatmap displaying the Pearson correlation coefficients between imputed, median-normalized protein intensities across samples, illustrating overall consistency in replicate samples. Bottom Panel**:** Principal component analysis (PCA) plot showing the clustering of different treatment groups. Variability within groups is noted, potentially due to the short treatment time, which may limit the magnitude of large-scale proteomic changes. Note: Gm-GFP WNK1 cells without sorbitol, Gp-GFP WNK1 cells with sorbitol, Hm-WT WNK1 cells without sorbitol and Hp-WT WNK1 cells with sorbitol. **(C)** Volcano plots highlighting proteins enriched in GFP-WNK1 KI HEK293 cells expressing FLAG-TurboID-aGFP6M treated with 0.5 mM biotin, with or without 0.5 M sorbitol. Protein intensities were normalized, imputed using a Gaussian distribution, and subjected to Benjamini-Hochberg correction for multiple hypothesis testing. Proteins with ≥2-fold enrichment and adjusted P-values <0.05 are annotated on the volcano plots, visualized using the Curtain tool. Proteins highlighted in green include the TurboID bait (WNK1) and its known interactors (WNK2, WNK3, SPAK, and OXSR1). Proteins highlighted in red are sorbitol-specific differential interactors of WNK1. **(D)** Western blot analysis of streptavidin pull-down fractions from GFP-WNK1 TurboID cells. Biotinylation of NRBP1, TSC22D2, TSC22D4, and ELOB is observed exclusively following hypertonic stress, validating their stress-specific enrichment. **(E)** Box plots depicting the median protein intensities of significantly enriched hits from the volcano plot in (C) and from Figure 1(D).

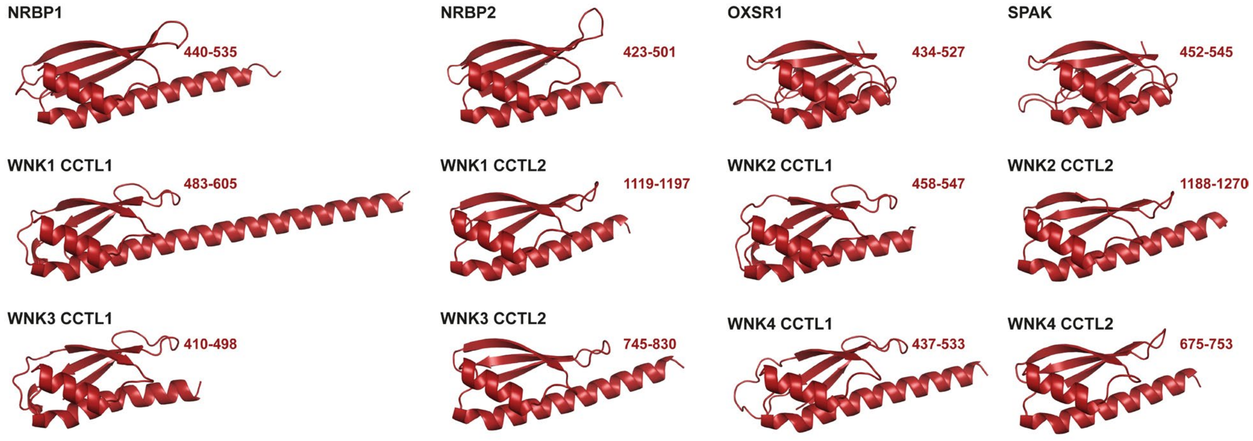

Fig. S2. Structural Analysis of CCT and CCTL Domains in signaling Proteins Structures of the CCT and CCTL domains of NRBP1, NRBP2, OXSR1, SPAK, WNK1, WNK2, WNK3, and WNK4, as derived from full-length AlphaFold 3 models.

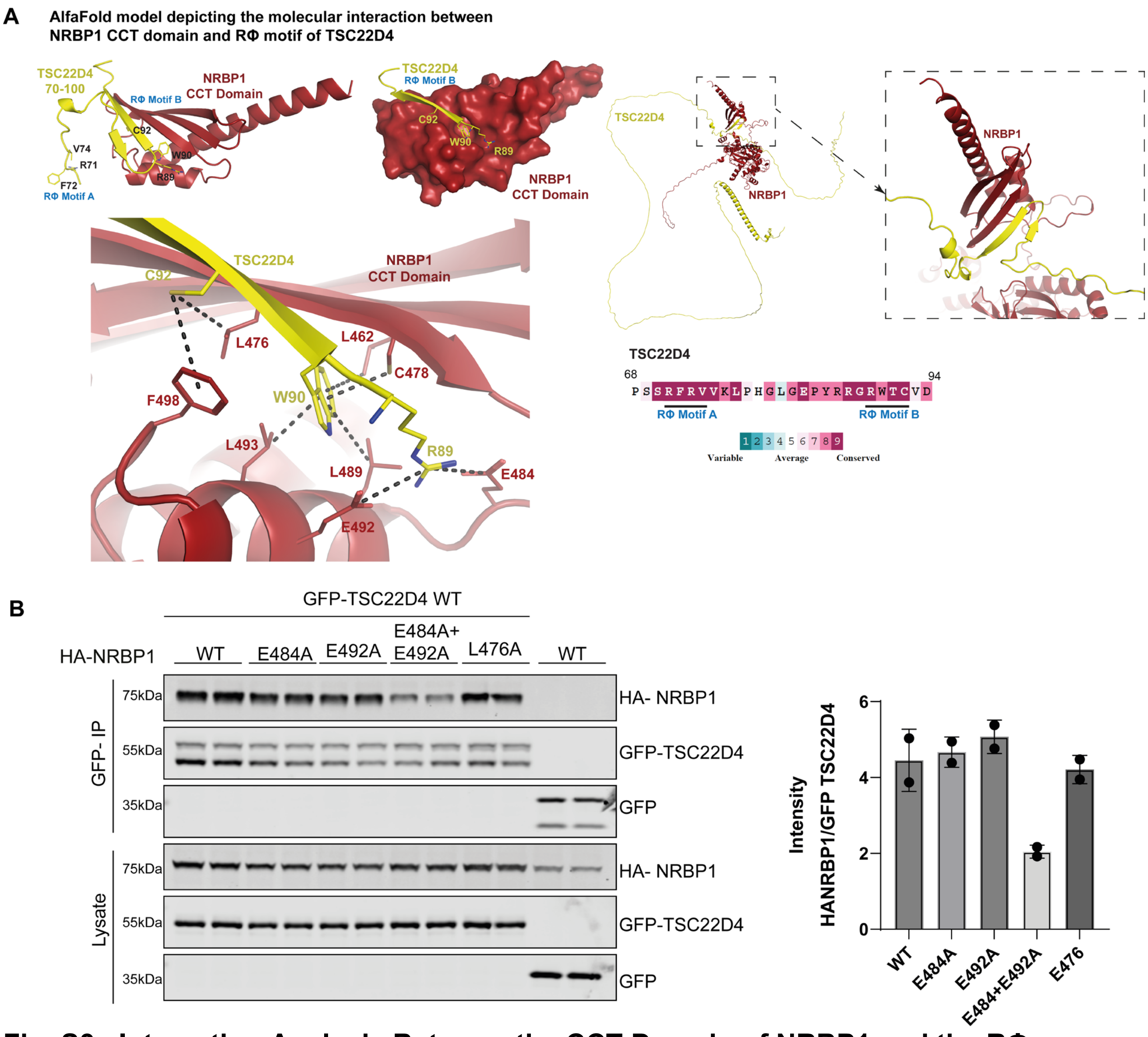

Fig. S3. Interaction Analysis Between the CCT Domain of NRBP1 and the RΦ Motif B of TSC22D4

(A) Detailed model of the interaction between the CCT domain of NRBP1 and the RΦ motif B of TSC22D4, extracted from a full-length AlphaFold 3 prediction. Trp90 of TSC22D4's RΦ motif B binds within a hydrophobic pocket of the CCT domain of NRBP1, formed by Leu462, Leu464, Leu476, Cys478, Leu489, Leu493, Leu496, and Phe498. Additional hydrophobic interactions are observed between Cys92 of TSC22D4 and Leu476 and Phe498 of NRBP1. Salt bridges are formed between Arg89 of TSC22D4 and Glu484 and Glu492 of NRBP1. ConSurf analysis (*84*) shows high conservation of RΦ motifs A and B in TSC22D4. **(B)** Co-immunoprecipitation of GFP-TSC22D4 WT and HA-NRBP1 mutants (E484A, E492A, and E476A) in HEK 293 cells after 36 h of co-transfection. Interaction was studied by GFP immunoprecipitation,

followed by western blotting. Right: Densitometric analysis of the western blots. Data represents the result of two independent experiments (*N=2*) with two technical replicates (n=2) for each.

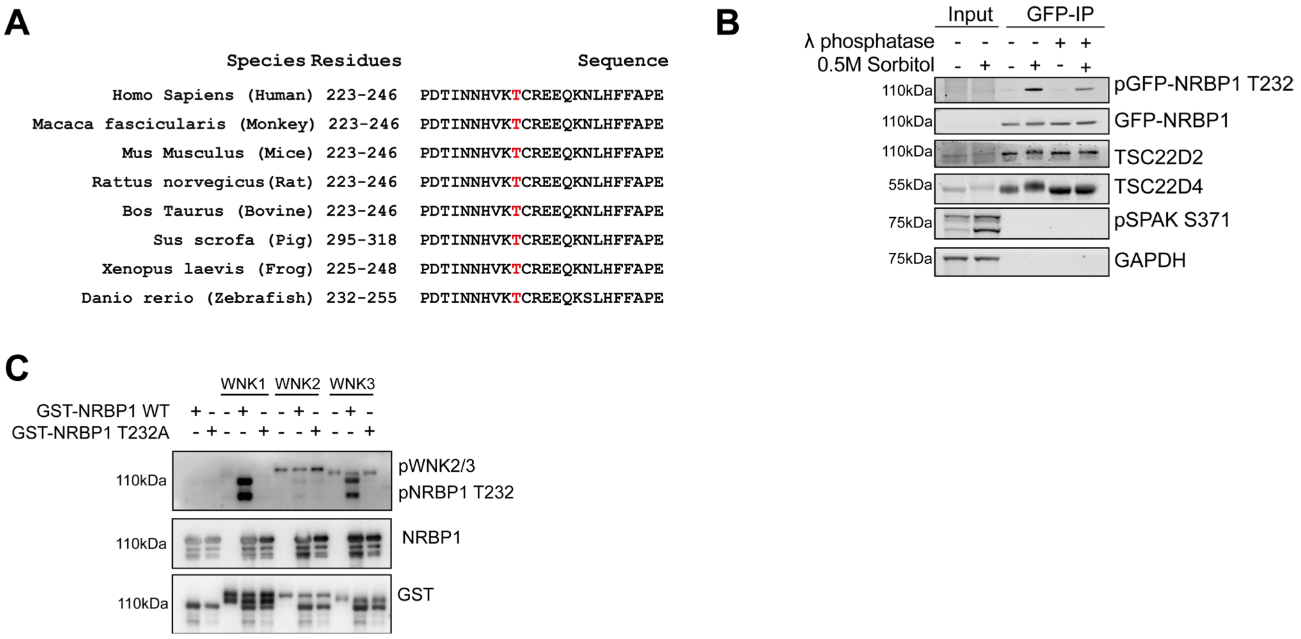

Fig. S4. Phosphorylation of NRBP1 and Its Interaction with TSC22D2/4
(A) Sequence alignment of the activation T-loop of NRBP1 from different species, performed using the MUSCLE tool (*85*), showing the evolutionary conservation of Thr232 (highlighted in red, numbering based on human sequence). **(B)** GFP-NRBP1-TSC22D2/4 complex was immunoprecipitated post-sorbitol treatment, and the immunoprecipitate was subjected to λ-phosphatase treatment to validate whether the observed TSC22D4 upshift is due to phosphorylation. **(C)** *In vitro* kinase assay with GST-tagged WNK1, WNK2, WNK3 kinases, and GST-tagged NRBP1 (WT & Thr232A mutant), showing that NRBP1 Thr232 is phosphorylated by WNK1 and WNK3. Results from n=2 are shown as a representative experiment.

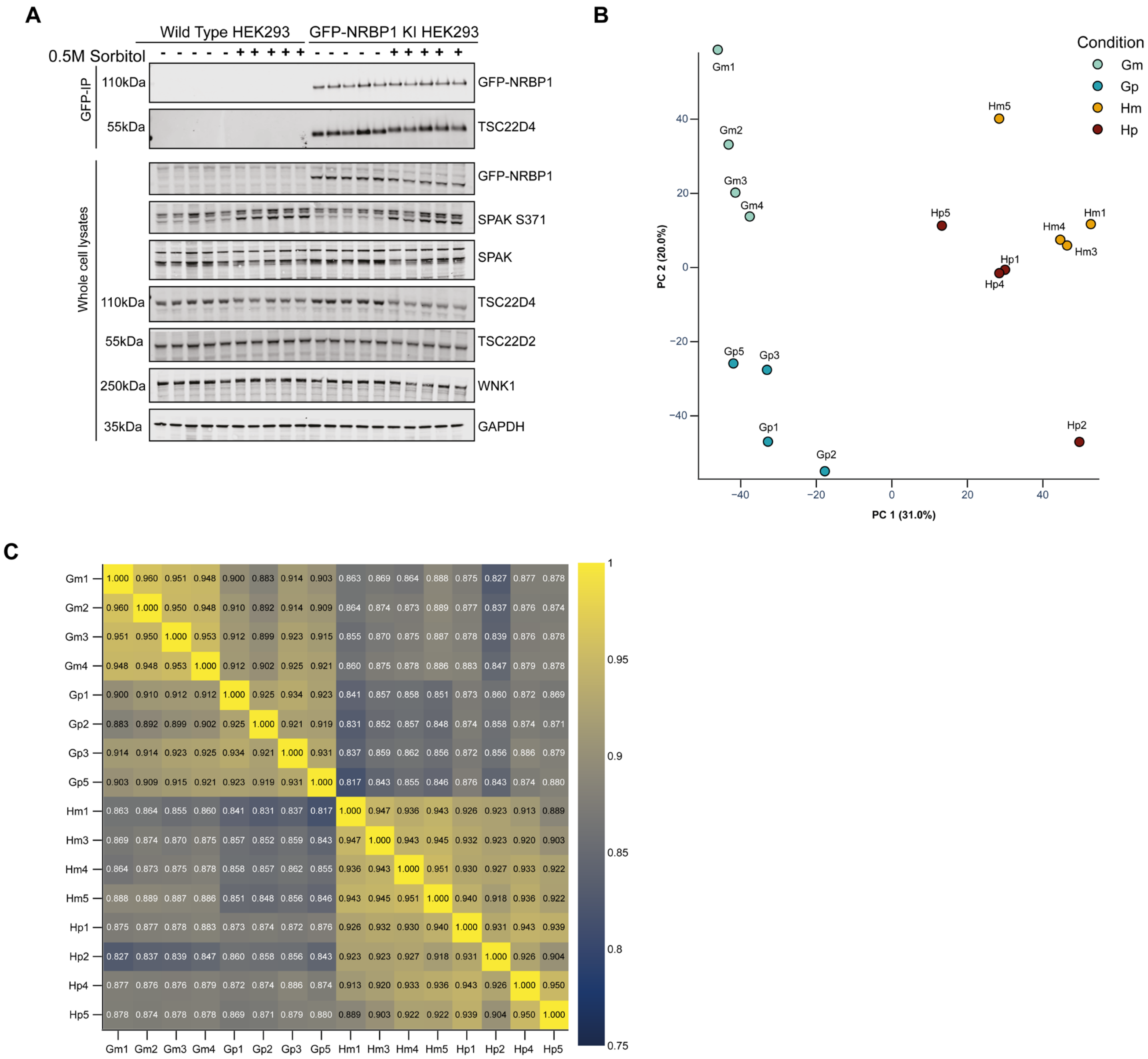

Fig. S5. Validation and Analysis of NRBP1 Interactors
(A) Immunoblots showing the activation of the WNK pathway and successful immunoprecipitation of GFP-NRBP1 for the mass spectrometry (MS) experiment described in Figure 5. n=5 technical replicates for each group. (B) Principal Component Analysis (PCA) plot showing the clustering of different treatment groups.
(C) Heatmap displaying the Pearson correlation coefficients between the imputed, median-normalized protein intensities across each sample. Note: Gm-GFP NRBP1 cells without sorbitol, Gp-GFP NRBP1 cells with sorbitol, Hm-WT NRBP1 cells without sorbitol and Hp-WT NRBP1 cells with sorbitol.

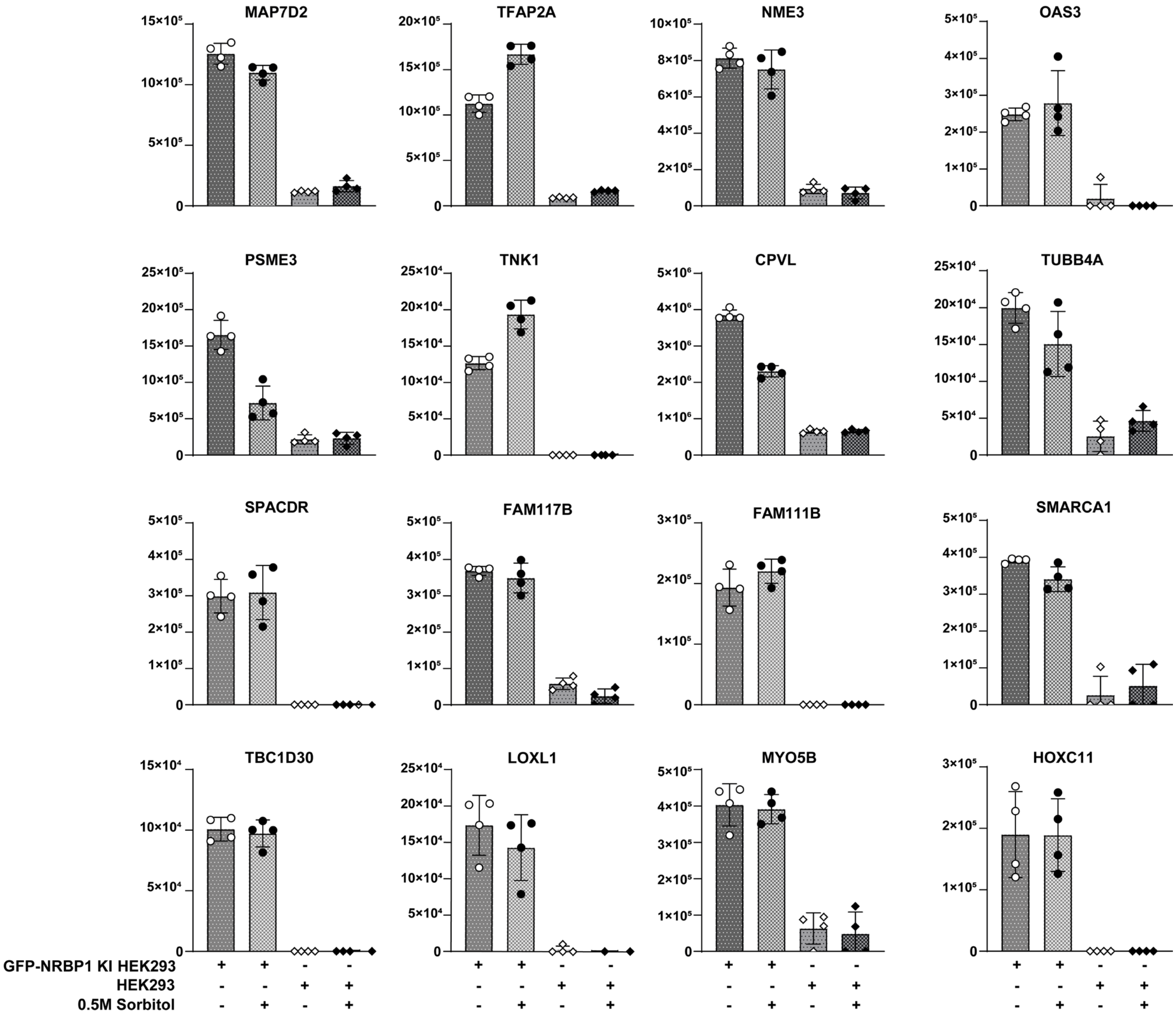

Fig. S6. Analysis of NRBP1 Interactors
Box plots showing the protein median intensities of selected NRBP1 interactors identified from the volcano plot in Figure 5B.

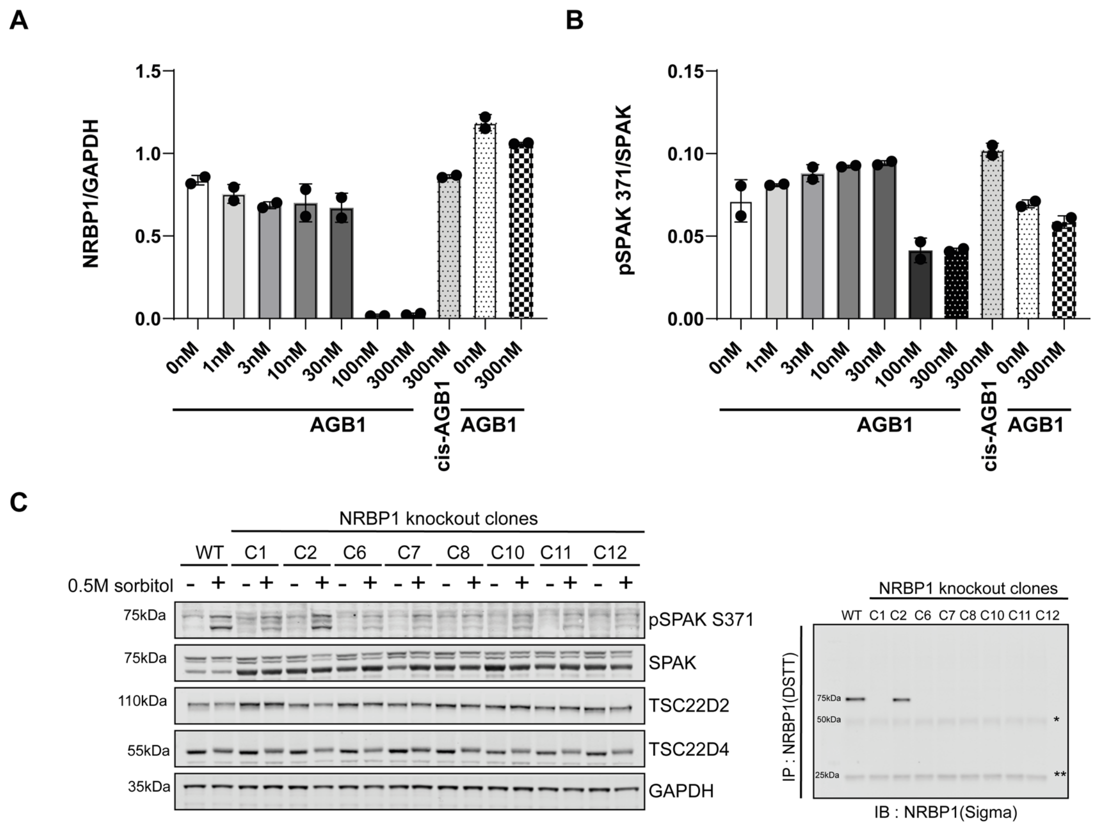

Fig. S7. Analysis of NRBP1 and SPAK Phosphorylation
(A & B) Densitometric analysis of NRBP1 and pSPAK levels, respectively, from the immunoblot in Figure 7A. **(C) Left Panel**: Effect of NRBP1 knockout (KO) on SPAK phosphorylation was analyzed by immunoblotting following 0.5 M sorbitol treatment for 30 min in 7 NRBP1 knockout HEK293 clones. **Right Panel**: Validation of NRBP1 knockout by endogenous immunoprecipitation and immunoblotting.

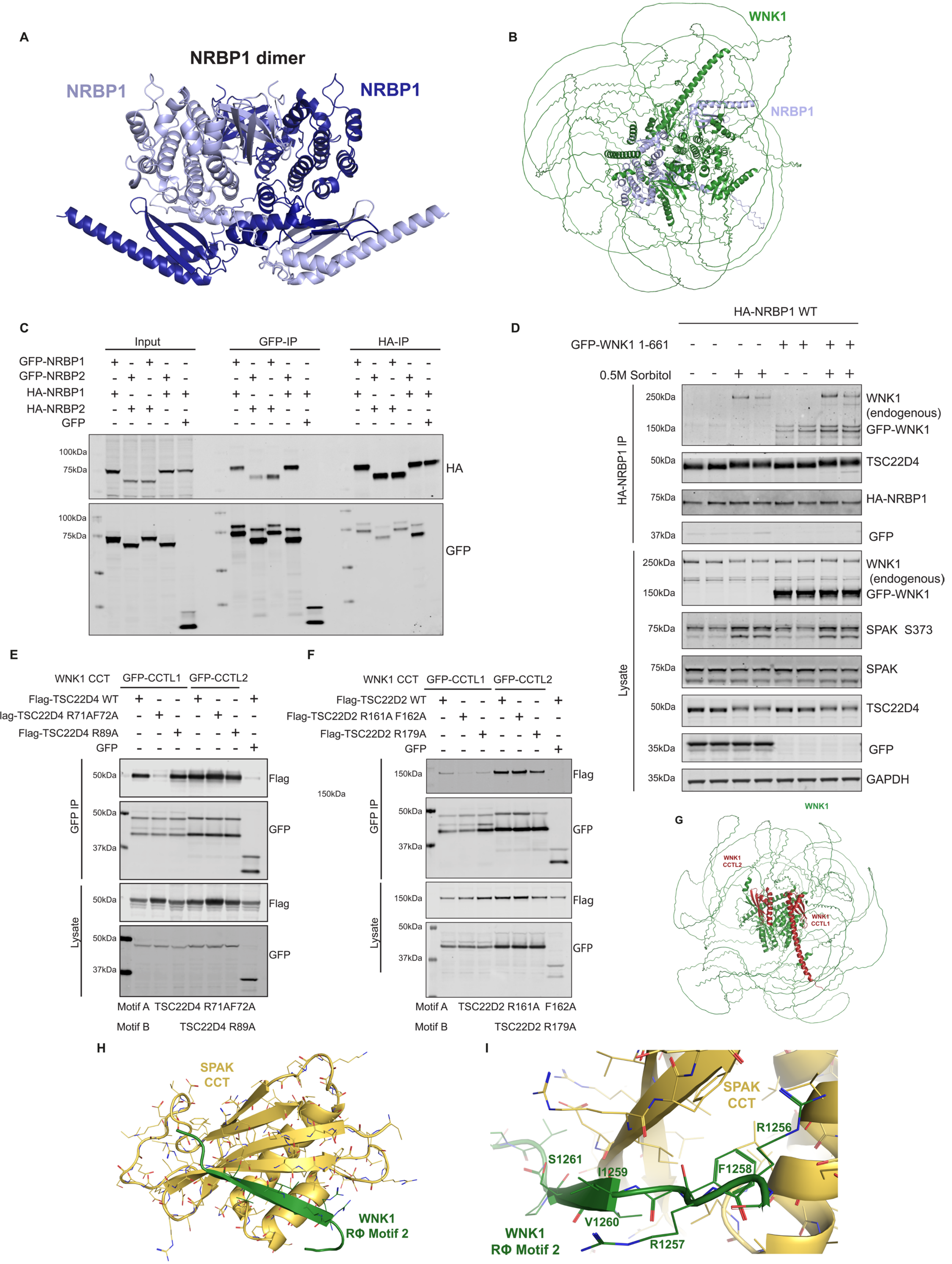

A
NRBP1 dimer
NRBP1
NRBP1
B
WNK1
NRBP1
C
Input
GFP-IP
HA-IP
GFP-NRBP1
GFP-NRBP2
HA-NRBP1
HA-NRBP2
GFP
100kDa
75kDa
HA
100kDa
75kDa
GFP
D
HA-NRBP1 WT
GFP-WNK1 1-661
0.5M Sorbitol
HA-NRBP1 IP
250kDa
150kDa
WNK1
(endogenous)
GFP-WNK1
50kDa
TSC22D4
75kDa
HA-NRBP1
37kDa
GFP
Lysate
250kDa
150kDa
WNK1
(endogenous)
GFP-WNK1
75kDa
SPAK S373
75kDa
SPAK
50kDa
TSC22D4
35kDa
GFP
35kDa
GAPDH
E
WNK1 CCT
GFP-CCTL1
GFP-CCTL2
Flag-TSC22D4 WT
Flag-TSC22D4 R71AF72A
Flag-TSC22D4 R89A
GFP
GFP IP
50kDa
Flag
50kDa
37kDa
GFP
Lysate
50kDa
Flag
50kDa
37kDa
GFP
Motif A TSC22D4 R71AF72A
Motif B TSC22D4 R89A
F
WNK1 CCT
GFP-CCTL1
GFP-CCTL2
Flag-TSC22D2 WT
Flag-TSC22D2 R161A F162A
Flag-TSC22D2 R179A
GFP
150kDa
GFP IP
150kDa
Flag
50kDa
37kDa
GFP
Lysate
150kDa
Flag
50kDa
37kDa
GFP
Motif A TSC22D2 R161A F162A
Motif B TSC22D2 R179A
G
WNK1
WNK1
CCTL2
WNK1
CCTL1
H
SPAK
CCT
WNK1
RΦ Motif 2
I
SPAK
CCT
R1256
S1261
I1259
F1258
V1260
R1257
WNK1
RΦ Motif 2

Fig. S8. AlphaFold 3 Models of NRBP1 and WNK1 Complexes
(A) AlphaFold 3 model of the NRBP1 homodimer. **(B)** AlphaFold 3 model of WNK1 in complex with NRBP1. **(C)** The homodimerization of NRBP1 and its heterodimerization with NRBP2 was studied by co-expressing the indicated GFP and HA tagged version of these proteins for 36 h, followed by GFP and HA-immunoprecipitations and immunoblotting with the indicated antibodies. **(D)** GFP-WNK1 1-661 was expressed in HEK293 cells stably expressing HA NRBP1. 36 h post transfection the HEK293 cells were treated ± sorbitol for 30 min followed by HA immunoprecipitation and immunoblotting with the indicated antibodies **(E & F)** GFP tagged WNK1 CCTL1 (residues 450 to 600) and CCTL2 domains (residues 1056-1217) of WNK1 were co-expressed in HEK293 cells with the indicated wild type (WT) and mutant versions of Flag tagged TSC22D2 (**E**) and Flag tagged TSC22D4 (**F**) for 36 hrs and their interaction was studied by GFP immunoprecipitation followed by immunoblotting for Flag and GFP. The experiment was repeated twice. **(G)** AlphaFold 3 model of full-length WNK1 with CCTL domains highlighted in red. **(H+I)** AlphaFold 3 model of the WNK1 RΦ-Motif-2 in complex with SPAK CCT domains.

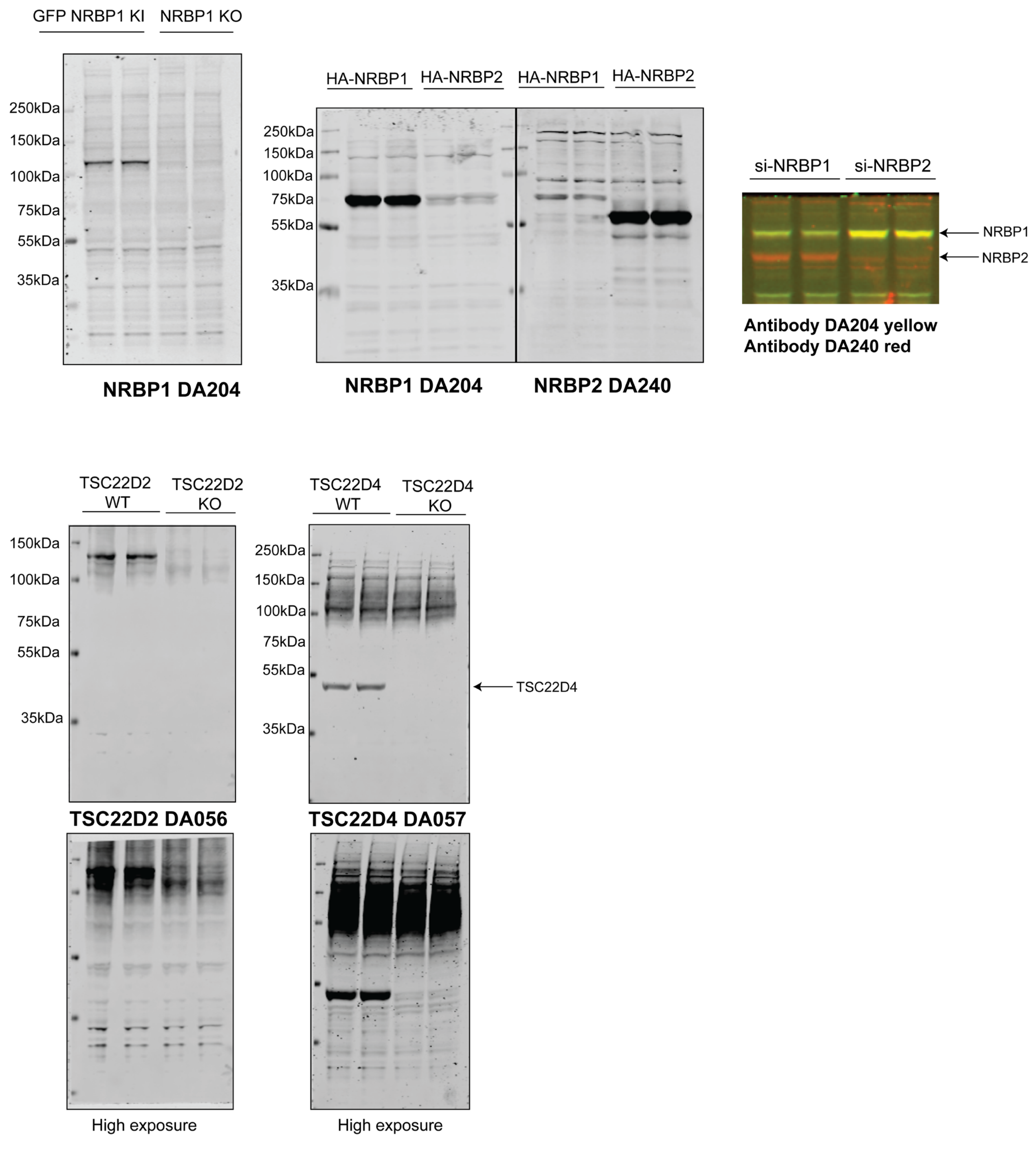

Fig. S9. Characterization of sheep polyclonal NRBP1, NRBP2, TSC22D2 and TSC22D4 antibodies

The specificity of the NRBP1 (residues 1 – 535, human sequence, DA204), NRBP2 (residues 1-509, human DA240), TSC22D2 (residues 4 – 780, human sequence, DA056) and TSC22D4 (residues 1 – 395, human sequence, DA057) sheep polyclonal antibodies raised for this study, was assessed by immunoblotting analysis employing wild type and the indicated knock-out HEK293 cells for NRBP1, TSC22D2 and TSC22D4. For the

NRBP1 and NRBP2 antibodies, validation was also performed by overexpression of HA-NRBP1 and HA-NRBP2 in HEK293 cells as well as by performing siRNA knockdown of NRBP1 or NRBP2. For siRNA transfection $5x10^5$ cells/well HEK3293 cells were seeded in 6 well plate for 24hrs prior to transfection. The transfection was carried out with Lipofectamine RNAiMAX and 12.5 pmol of siRNA/well as per the manufacturer's protocol. Post 72 hrs transfection the cells were harvested using 1% NP-40 lysis buffer and immunoblotted. The siRNA used was ON-TARGET plus Human NRBP2 (340371) siRNA SMARTpool and the targeted sequence were UGGAGAGAAGCGAGGACAA, CUUCAUGGAGCUGGACAAA, GGGUCACAGAGGAGGCCAU, GAGAUGGCUGUACUGGAAA.
